# Antarctic Hairgrass Rhizosphere Microbiomes: Microscale Effects Shape Diversity, Structure, and Function

**DOI:** 10.1264/jsme2.ME21069

**Published:** 2022-06-15

**Authors:** Ievgeniia Prekrasna, Mariia Pavlovska, Natalia Miryuta, Artem Dzhulai, Evgen Dykyi, Peter Convey, Iryna Kozeretska, Tymur Bedernichek, Ivan Parnikoza

**Affiliations:** 1 State Institution National Antarctic Scientific Center, 16 Taras Shevchenko Blvd., 01601, Kyiv, Ukraine; 2 British Antarctic Survey, NERC, High Cross, Madingley Road, Cambridge, CB3 0ET, UK; 3 Department of Zoology, University of Johannesburg, PO Box 524, Auckland Park 2006, South Africa; 4 M.M. Gryshko National Botanical Garden, 1 Timiryazev Str., 01014, Kyiv, Ukraine; 5 Institute of Molecular Biology and Genetics, 150 Zabolotnogo Str., 03143, Kyiv, Ukraine; 6 National University of “Kyiv-Mohyla Academy”, st. Skovorody, 2, Kyiv, 04070, Ukraine; 7 National University of Life and Environmental Sciences of Ukraine, 15 Heroiv Oborony Str., 03041, Kyiv, Ukraine

**Keywords:** rhizosphere microbiome, Antarctic vascular plants, microbial diversity, microbial functional repertoire

## Abstract

The rhizosphere microbiome of the native Antarctic hairgrass *Deschampsia antarctica* from the central maritime Antarctic was investigated using 16S RNA metagenomics and compared to those of the second native Antarctic plant *Colobanthus quitensis* and closely related temperate *D. cespitosa*. The rhizosphere microbial communities of *D. antarctica* and* D. cespitosa* had high taxon richness, while that of *C. quitensis* had markedly lower diversity. The majority of bacteria in the rhizosphere communities of the hairgrass were affiliated to *Proteobacteria*, *Bacteroidetes*, and *Actinobacteria*. The rhizosphere of *C. quitensis* was dominated by *Actinobacteria*. All microbial communities included high proportions of unique amplicon sequence variants (ASVs) and there was high heterogeneity between samples at the ASV level. The soil parameters examined did not explain this heterogeneity. Bacteria belonging to *Actinobacteria*, *Bacteroidetes*, and *Proteobacteria* were sensitive to fluctuations in the soil surface temperature. The values of the United Soil Surface Temperature Influence Index (UTII, *I^t^_i_*) showed that variations in most microbial communities from Galindez Island were associated with microscale variations in temperature. Metabolic predictions *in silico* using PICRUSt 2.0, based on the taxonomically affiliated part of the microbiomes, showed similarities with the rhizosphere community of *D. antarctica* in terms of the predicted functional repertoire. The results obtained indicate that these communities are involved in the primary processes of soil development (particularly the degradation of lignin and lignin-derived compounds) in the central maritime Antarctic and may be beneficial for the growth of Antarctic vascular plants. However, due to the limitations associated with interpreting PICRUSt 2.0 outputs, these predictions need to be verified experimentally.

Antarctica contains some of the world’s most pristine and severe environments due to its geographical isolation and the effects of physical stressors, including low temperatures, intense UV radiation, and desiccation (Convey *et al.*, 2014, 2018). At least 99.6% of terrestrial Antarctica is permanently covered by ice and snow, with the majority of ice-free areas being located in coastal regions, particularly in the western Antarctic Peninsula and Scotia Arc archipelagoes (Terauds *et al.*, 2012; Burton-Johnson *et al.*, 2016). Only a small fraction of this ice-free area is visibly colonized by macroscopic algae, bryophytes, and vascular plants (Alberdi *et al.*, 2002; Gray *et al.*, 2020).

Two vascular plants are native to the maritime Antarctic, the Antarctic hairgrass *Deschampsia antarctica* Ė. Desv. and the pearlwort *Colobanthus quitensis* (Kunth) Bartl. Both generally occur in mixed communities with bryophytes and lichens and in relatively small patches (Smith, 2003; Bokhorst *et al.*, 2007; Parnikoza *et al.*, 2009). They also occur in coastal areas along the length of the western Antarctic Peninsula, reaching northern Alexander Island (69°S), as well as in southern and Andean South America (Convey *et al.*, 2011). The very limited number of vascular plant species in the maritime Antarctic has led to them being of scientific interest in the context of their colonization of the region (Smith, 2003; Parnikoza *et al.*, 2007; 2011). To date, no adaptations exclusive to the Antarctic have been discovered in either species (Mantovani and Vieira, 2000; Barcikowski *et al.*, 2001; Gielwanowska and Szczuka, 2005). Their ability to survive in Antarctica appears to be attributed to interactions with other organisms that have co-evolved with these plants, and not to specific mechanisms or adaptations (Parnikoza *et al.*, 2012; Ozheredova *et al.*, 2015). The interaction of *D. antarctica* with birds assists in its dispersal (Parnikoza *et al.*, 2018). Other advantages may be obtained through interactions with prokaryotes. Prokaryotes are important for plant survival and are the dominant biomass component of Antarctic terrestrial ecosystems (Wynn-Williams, 1990; Pointing *et al.*, 2009).

Prokaryotes are recognized as the primary drivers of nutrient cycling in soils and may be impacted by a number of factors, including pH, total carbon, nitrogen, moisture, and other edaphic parameters (Chong *et al.*, 2009; 2010; 2012; Ganzert *et al.*, 2011; Stomeo *et al.*, 2012; Wang *et al.*, 2015). Temperature is considered to be an important factor influencing the diversity of bacterial communities in maritime Antarctic soils (Dennis *et al.*, 2019). Prokaryotes living in the vicinity of a plant root are often associated with plant development and health, productivity, and defense against pathogens (Berg and Smalla, 2009; Lugtenberg and Kamilova, 2009). Their beneficial effects on plant development include facilitating resource acquisition, modulating phytohormone levels, and synthesizing antibiotics and lytic enzymes. Rhizosphere bacteria increase the bioavailability of some of the nutrients required by plants. Soil bacteria, such as *Rhizobium, Bradyrhizobium*, and *Azospirillum*, are involved in nitrogen fixation and its supply to plants (Glick *et al.*, 2007; Lugtenberg and Kamilova, 2009). Rhizosphere bacteria are also capable of solubilizing phosphorus and iron compounds through the synthesis of phosphatases, phytases, phosphatases and C-P lyases, organic acids, and iron-mobilizing proteins known as siderophores (Rodríguez‍ ‍and‍ ‍Fraga,‍ ‍1999;‍ ‍Richardson, 2001;‍ ‍Rodriguez‍ ‍*et‍ ‍al.*,‍ ‍2004).‍ ‍Some bacteria-derived compounds directly promote plant growth, such as phytohormones (*e.g.* indole acetic acid [IAA]), 1-aminocyclopropane-1-carboxylate (ACC) deaminase, 2,3-butanediol, and acetoin (Glick *et al.*, 2007; Dodd *et al.*, 2010; Spaepen and Vanderleyden, 2011). Bacteria are also capable of indirectly affecting plants through the synthesis of antibiotics and lytic enzymes that act against pathogenic organisms (Whipps, 2001; Glick, 2012).

A number of studies have examined Antarctic vascular plant root-associated microorganisms at various localities in one specific region of the northern maritime Antarctic, the South Shetland Islands. Various bacterial strains (*Pseudomonas* sp., *Flavobacterium* sp., *Arthrobacter *sp., and *Clostridium* sp.) have been isolated from the rhizosphere of *D. antarctica*, some of which possess plant-beneficial properties, such as phosphorus solubilization and plant growth promotion (Barrientos-Díaz *et al.*, 2008; Berríos *et al.*, 2013; Peixoto *et al.*, 2016). Members of the genera *Rhodococcus*, *Enterobacter*, *Devosia*, *Burkholderia*, *Arthrobacter*, and *Planococcus* isolated from the rhizosphere of Antarctic vascular plants improved their ability to tolerate salt stress (Gallardo-Cerda *et al.*, 2018). In addition to culture-dependent studies, 454 pyrosequencing has been applied to examine the composition of microbial communities in the rhizosphere soil of *D. antarctica* and *C. quitensis* (Teixeira *et al.*, 2010) on King George Island. This study showed similar patterns of bacterial diversity between the two plant species, and the genera *Bifidobacterium*, *Arcobacter*, and *Faecalibacterium* appeared to be dominant in the rhizosphere microbiomes. Roesch *et al.* (2012) studied microbiomes from soil taken from a number of plant communities on the Keller Peninsula (King George Island), and identified pH as the best predictor of the microbial community structure. The recent application of shotgun metagenomics to investigate the structure and functions of the rhizosphere microbial communities of plants growing on Livingstone Island identified *Sporosarcina* and *Chryseobacterium* as the most abundant genera (Molina-Montenegro *et al.*, 2019).

The wider Antarctic distributions of these two plant species encompass localities that vary widely in their edaphic and microclimatic conditions. Even at relatively small spatial scales, habitats differ widely in the time since deglaciation, the underlying geology, soil development, and overall vegetation. To extend the generality of the few studies performed on Antarctic rhizosphere microbiomes, the geographic coverage of the plant populations examined needs to be expanded. *C. quitensis* colonizes similar biotopes and experiences the same abiotic stresses as *D. antarctica*, which encourages similarities in the composition of the rhizosphere. On the other hand, plant species may themselves be an important factor shaping the rhizosphere community. Comparisons of rhizosphere microbiome compositions with the second native Antarctic plant *C. quitensis* and other closely related non-Antarctic hairgrass species, such as *D. cespitosa*, are also appropriate. Therefore, the present study focused on the populations of Antarctic hairgrass that are present in the Argentine Islands and Palmer Archipelago (Parnikoza *et al.*, 2007; 2018) in the central maritime Antarctic, which is approximately 400‍ ‍km south of the South Shetland Islands. The aim of this study was to describe the diversity, taxonomic composition, and potential functional repertoire, including plant growth-promoting capacity, of the rhizosphere microbial communities associated with Antarctic hairgrass growing on different parts of Galindez and Anvers Islands, and compare them with the rhizosphere microbiomes of *C. quitensis* and *D. cespitosa*.

## Materials and Methods

### Sampling sites and sampling procedure

**Fig. 1.**
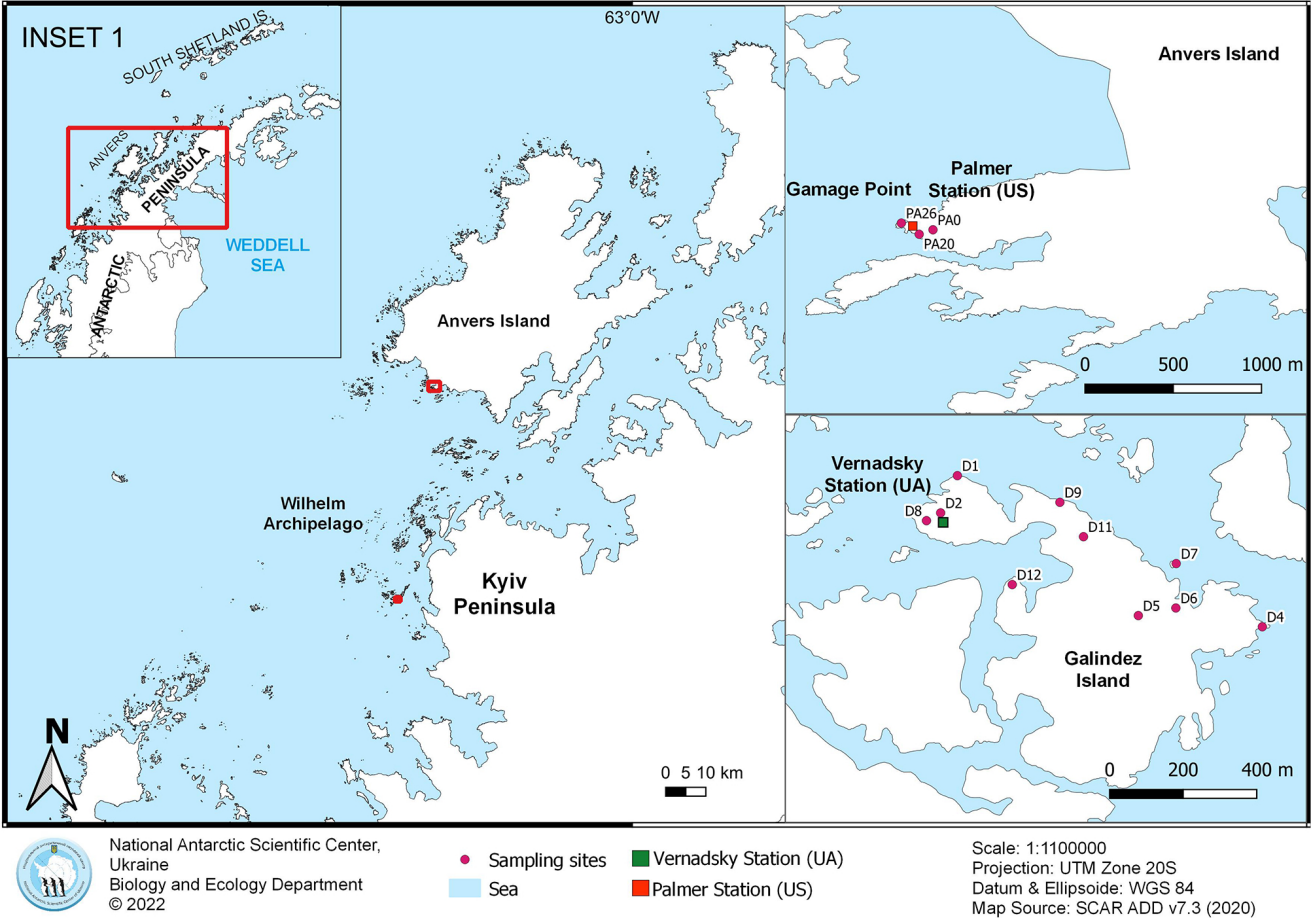
Location of sampling sites on Galindez and Anvers Islands, western Antarctic Peninsula, the maritime Antarctic.

Sampling was conducted in February 2018, during the 23^rd^ Ukrainian Antarctic Expedition, on Galindez Island (Argentine Islands) near the Ukrainian Vernadsky Antarctic Station and on Anvers Island at Gamage Point near the US Palmer Station (Palmer Archipelago). The Argentine Islands and Palmer Archipelago are located on the coastal shelf of the western Antarctic Peninsula (Fig. 1 and Table 1). Rhizosphere soil (soil aggregated around plant roots) was collected under nine populations of *D. antarctica* on Galindez Island (Table 1: samples D1, D2, D4, D5, D7, D8, D9, D11, and D12) and three populations on Anvers Island (Table 1: samples PA0, PA20, and PA26; Fig. 1). One sample of the closely related species *D. cespitosa* (L.) P. Beauv. was collected in Punta Arenas, Chile (-53.163967, -70.896633) for comparison with the microbiome of the Antarctic hair grass rhizosphere; this sample was obtained within the project FONDECYT 1181745 (the influence of penguin colonies on the development of tundra communities in the Antarctic Peninsula) in collaboration with Dr. A. Casanova-Katny. Fourteen samples of rhizosphere soil were used in ana­lyses (Table 1). In an initial comparison of the rhizosphere of the Antarctic hair grass with the second native Antarctic vascular plant, one sample from a single population of *C. quitensis* on Galindez Island (Table 1: sample D6) was collected. *C. quitensis* has a very limited occurrence in the Argentine Islands.

The vegetation of the sampling locations included communities of the bryophyte carpet and mat subformation with *D. antarctica* (Table 1). The cover of *D. antarctica* at specific sampling locations varied between 1 and 15% at the time of collection (Table 1).

The main types of soils on Galindez and Anvers Islands (leptosols) have undeveloped profiles with a shallow active layer (4–20‍ ‍cm), are present in small niches between rocks, and provide limited opportunities for root growth (Fig. S2). At each sampling site, the upper layer of soil under the plant was removed, and soil surrounding the root system (1–2‍ ‍mm around the root) was collected in a sterile 15-mL Falcon tube. DESS solution (Pavlovska *et al.*, 2021) was added to the sample at a volume ratio of 3:1 for the preservation of prokaryotic cells and DNA during transportation. Samples were stored at –20°C and DNA was extracted within one month of collection.

### Measurement of soil chemical parameters

Primary soils in Antarctica have low depth and typically occupy small areas in crevices between rocks and under vegetation cover. Since the intensive sampling of soil may lead to the destruction of the ecosystem, we limited our sampling and used existing data (Parnikoza *et al.*, 2016) on soil chemical parameters from sampling sites on Galindez Island. Parameters included total organic carbon, pH, the total contents (%) of N, P_2_O_5_, and K_2_O, and the concentrations (mg kg^–1^) of Cu, Ni, Pb, Cd, Zn, Mn, and Fe. The same parameters were measured for samples obtained on Anvers Island. Briefly, soil samples were air-dried, ground using an agаte mortar and pestle, and sieved through a 2-mm sieve. The samples obtained were divided into sub-samples and used in further ana­lyses. pH was measured in a 1:5 (volume fraction) suspension of soil in water and in 1 M KCl solution according to ISO 10390:2005 on a SevenMulti Benchtop Meter (Mettler-Toledo). In further ana­lyses, sub-samples were ground using the mortar and pestle and sieved through a 0.25-mm sieve. The total organic carbon content was measured using the wet combustion method (ISO 14235: 1998). The absorbance of the solutions obtained was measured spectrophotometrically using a SPEKOL 2000 spectrophotometer (Analytik Jena). Total nitrogen content was assessed using the modified Kjeldahl method (ISO 11261:1995 Soil). The microwave-assisted digestion of soil samples in concentrated HNO_3_ was performed in DAP-60K vessels in MWS-2 (Berghoff). The contents of P, K, S, and other chemical elements were measured using an ICP-OES Spectrometer iCAP 6300 Duo (Thermo Fisher Scientific). Double distilled water was used for all steps of soil ana­lyses. Chemical content is expressed as the proportion of dry matter.

### Measurement of the soil surface temperature

HOBO UA-002-64 loggers were installed between plant clumps in the populations of *D. antarctica* (D1, D2, D4, D5, D7, D8, D9, D11, and D12) and population D6 (*D. antarctica* and *C. quitensis* with bryophytes) on Galindez island. The temperature of the soil surface was recorded every 30‍ ‍min between 09.04.2017 and 07.04.2018.

### DNA extraction and sequencing

DNA was extracted from soil using the Quick-DNA Fecal/Soil Microbe Kit (Zymo Research) following the manufacturer’s instructions. The concentration and purity of DNA were quantified using a Nanodrop ND-1000 spectrophotometer (Thermo Fisher Scientific). The 16S rRNA gene V4 region was sequenced by the Illumina MiSeq platform (MR DNA) using the bacterial primer pair S-D-Bact-0341-b-S-17 and S-D-Bact-0785-a-A-21 (Klindworth *et al.*, 2013), with a barcode on the forward primer. Prior to sequencing, single-step 28-cycle PCR was performed using the HotStarTaq Plus Master Mix Kit (Qiagen) under the following thermal conditions: 94°C (3‍ ‍min), 28 cycles of 94°C for 30‍ ‍s, 53°C for 40‍ ‍s, and 72°C for 1‍ ‍min, with final elongation at 72°C for 5‍ ‍min. PCR products were checked using a 2% agarose gel.

### Bioinformatics and statistical ana­lysis

Raw sequencing data were demultiplexed, denoised, quality filtered, and taxonomically assigned using the QIIME2 2019.7 pipeline (Bolyen *et al.*, 2019). Sequences shorter than 150 bp were discarded. There were 75,851 to 107,201 sequences per sample, with a total of 8,298 amplicon sequence variants (ASVs) identified after quality filtering. Taxonomy was assigned using the Greengenes 13_8 database. Shannon index values and the Bray-Curtis distance matrix were estimated with the “diversity core metrics” plugin in QIIME2 2019.7. Sequences were uploaded to the NCBI database under accession number PRJNA591781.

PICRUSt 2.0 (Douglas, G., *et al.* 2019. PICRUSt2: An improved and customizable approach for metagenome inference. biorxiv. https://doi.org/10.1101/672295) was used to estimate the abundance of gene families (KEGG orthologs, Enzyme Classification numbers) and MetaCyc ontology predictions contributed to a metagenome by prokaryotes identified using 16S rRNA sequencing. Conclusions about microbiome functions derived from PICRUSt 2.0 may be treated as hypotheses that require further in-depth validation through functional assays. PICRUSt 2.0 was run in QIIME2 2019.7 with the command “qiime picrust2 full-pipeline” with mp as a hidden-state prediction (HSP) method, and 2 as the maximum NSTI value.

Further ana­lyses were performed using R Studio 3.6.0 and the packages ‘biomformat’, ‘vegan’, and ‘EdgeR’. Shapiro-Wilk tests of normality were run to test the normality of alpha-diversity data.

The Kruskal-Wallis rank-sum test (*P*=0.05) was used to compare alpha-diversity data between groups and the abundance of prokaryotic taxa at the phylum and family levels between communities from Galindez and Anvers Islands. A general linear model with a negative binomial distribution (‘EdgeR’ package) was used to identify significant relationships between chemical factors and bacterial families in rhizosphere communities. *P*-values were corrected to account for multiple comparisons using the false discovery rate (q-value) method (Benjamini and Hochberg, 1995). Results with q-value <0.05 were considered to be significant. Only data for the rhizosphere microbiome of *D. antarctica* were considered for these calculations due to the lack of replication for other plant species.

A Mantel test was applied to assess the influence of temperature on taxon abundance in rhizosphere microbial communities. Pairwise comparisons were performed on the relative abundance of bacterial phyla spatial differences and average temperature spatial differences in December 2017, January 2018, and February 2018. These two matrices were compared using the Mantel test (Pollard, 1982; Legendre *et al.*, 2015). The temperature spatial differences matrix was also compared with the Weighted Unifrac Distance matrix calculated for the rhizosphere microbial communities from Galindez Island to estimate the impact of soil surface temperatures on microbial community compositions. The extreme grouping method was used to calculate the United Soil Surface Temperature Influence Index (UTII, *I^t^_i_*) for rhizosphere microbial communities as described by Miryuta *et al.* (2019).

## Results

### Soil chemical parameters

Carbon and nitrogen contents differed in soils from Galindez and Anvers Islands (Table 2). The input of bird guano through proximity to bird rookeries, along with decaying plant material, was responsible for the high soil carbon and nitrogen contents on Galindez Island. These soils had a neutral or slightly acidic pH, a high organic content with the initial stages of humification, and a high C:N ratio (Table 2). Soil from Anvers Island had lower carbon and nitrogen contents (Table 2) and a slightly acidic pH. No consistent patterns were observed in the contents of potassium, phosphorus, or trace elements, with high variations being noted across samples.

### Sequencing outputs and diversity of rhizosphere microbial communities

Illumina MiSeq identified between 75,851 and 107,201 sequences (Table 3) of the partial 16S rRNA gene per sample, with an average length of 251 bp. Based on rarefaction curves (Fig. S1), significantly higher estimates were not achievable by deeper sequencing. Therefore, we considered the study to have adequately covered abundant and rare ASVs in the communities examined.

A total of 8,298 ASVs were obtained after data filtering (Table 3). The rhizosphere microbiomes of *D. antarctica* growing on Galindez and Anvers Islands included very similar ASV numbers (H=0.03, *P*=0.86) and Shannon index values (H=0.03, *P*=0.86). Shannon index values indicated high taxon richness in these microbiomes and an even distribution of ASVs. The diversity of the microbiome from the rhizosphere soil of southern temperate *D. cespitosa* was similar to that obtained from *D. antarctica* (H=0.07, *P*=0.78). ASV numbers and the Shannon index value were both markedly lower for the rhizosphere microbial community of *C. quitensis*, even though the number of reads did not differ (Table 3). However, it was not possible to confirm that the difference in the diversity of the microbiomes of *D. antarctica* and *C. quitensis* was significant because there was only a single sample of the *C. quitensis* microbiome.

**Fig. 2.**
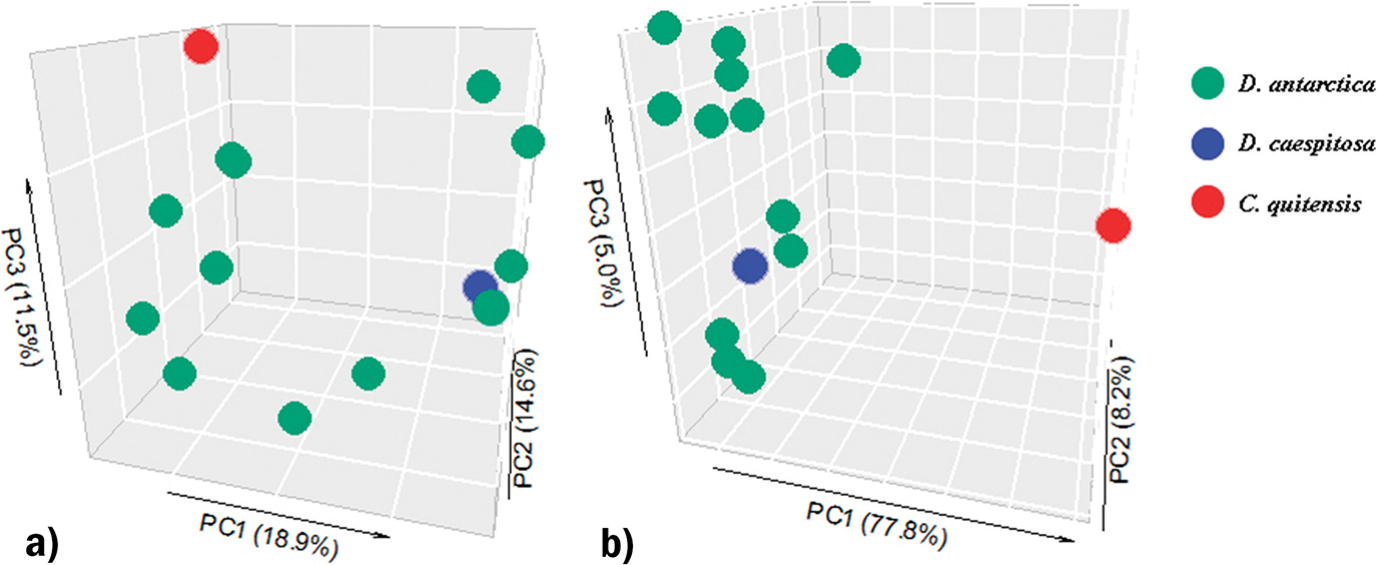
Principle coordinate ana­lyses based on the Bray-Curtis distance matrix of the ASV (A) and metabolic pathway (B) distribution across rhizosphere samples of 1—*Deschampsia antarctica*, Galindez Is.; 2—*D. antarctica*, Anvers Is.; 3—*Deschampsia cespitosa*, Punta Arenas; 4—*Colobanthus quitensis*, Galindez Is.

The measured soil parameters did not significantly affect Shannon index values. A principal coordinate ana­lysis (PCoA) based on the Bray Curtis distance matrix (Fig. 2A), calculated from the relative abundance of each ASV, showed dissimilarity between samples (Fig. 2A). This was attributed to the high percentage of unique ASVs in each sample, ranging between 19.6 and 63.3% (Table 3). Only six ASVs were shared between all samples of the soil microbiome of *D. antarctica*, and were affiliated with the genera *Arthrobacter*, *Psychrobacter*, *Staphylococcus*, *Clostridium*, and *Salinibacterium* and order *Myxococcales* (Table S1). Estimates of the numbers of shared ASVs were not higher in samples taken from the same location (*i.e.* from Galindez or Anvers Island). All shared ASVs were also present in the rhizospheres of *C. quitensis* and *D. cespitosa*, suggesting that these taxa are ubiquitous and not specific to *D. antarctica* or the Antarctic Peninsula region.

### Taxonomic assignment

ASVs were assigned to 37 prokaryotic phyla, 114 classes, 224 families, and 301 genera (Table S2). The proportions of unassigned sequences increased when lower taxonomic levels were considered, with approximately 4, 7, 41, and 76% of sequences remaining unclassified at the phylum, class, family, and genus levels, respectively. Since only a small proportion of ASVs were assigned at the generic level, we considered families as the lowest taxonomic level in subsequent ana­lyses.

**Fig. 3.**
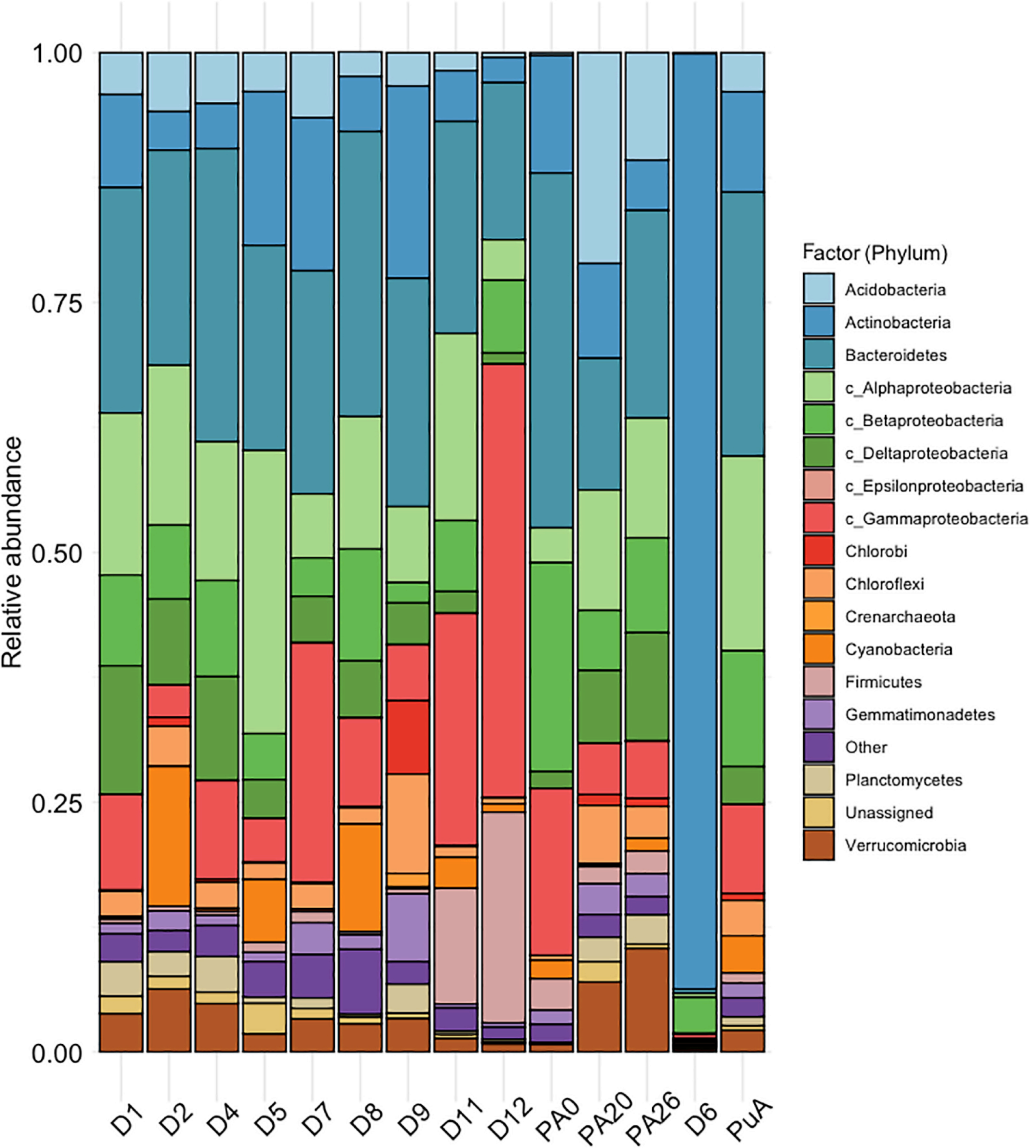
Taxonomic composition of microbial communities at the phylum level inhabiting rhizosphere soil. D1, D2, D4, D7, D8, D9, D11, and D12—*Deschampsia antarctica*, Galindez Is.; PA0, PA20, and PA26, - *D. antarctica*, Anvers Is.; D6—*Colobanthus quitensis*, Galindez Is.; PuA—*Deschampsia cespitosa*, Punta Arenas;

Among the 37 phyla detected, 11 accounted for 95–99% of the communities. *D. antarctica* rhizosphere microbiomes from Galindez and Anvers Islands had similar compositions at the phylum level (Fig. 3). A list of prokaryotic taxa identified in rhizosphere microbial communities is given in Table S2. *Proteobacteria* (42.6±10.6%), *Bacteroidetes* (22.7±4.1%), and *Actinobacteria* (9.0±6.1%) were the most abundant phyla in all *D. antarctica* rhizosphere communities. *Acidobacteria* (3.7±1.9%), *Cyanobacteria* (4.0±5.2%), *Firmicutes* (4.1±7.6%), *Gemmatimonadetes* (1.9±2.0%), *Verrucomicrobia* (3.2±1.7%), *Planctomycetes* (1.7±1.4%), *Chlorobi* (1.1±2.4%), and *Chloroflexi* (2.9±2.8%) were the other abundant phyla. Archaea represented only a small proportion of the population, mainly *Crenarchaeota* (0.2±0.6%). The only difference between microbiomes from Galindez and Anvers Islands was that *Acidobacteria* (10.7±10.4%) and *Verrucomicrobia* (6.0±4.9%) accounted for higher proportions of the community in the latter; however, the ana­lysis using the Kruskal-Wallis rank-sum test did not identify these differences as significant (H=1.03, *P*=0.3 and H=1.03, *P*=0.3, respectively). The *D. cespitosa* rhizosphere microbiome comprised similar bacterial phyla to that of *D. antarctica*, while that of *C. quitensis* (sample D6) was markedly different and strongly dominated by *Actinobacteria* (93.6%).

**Fig. 4.**
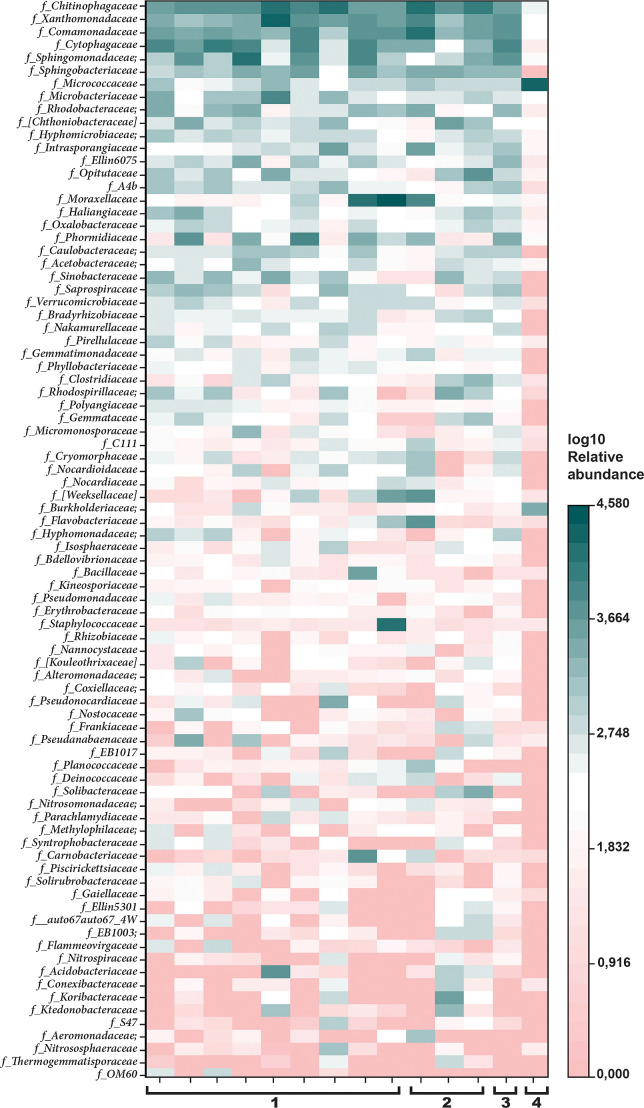
Relative (log_10_) abundance of most abundant families (contributing >0.05%) in rhizosphere soil of 1—*Deschampsia antarctica*, Galindez Is.; 2—*D. antarctica*, Anvers Is.; 3—*Deschampsia cespitosa*, Punta Arenas; 4—*Colobanthus quitensis*, Galindez Is.

Differences between microbial communities were more apparent at lower taxonomic levels. Fig. 4 shows the patchy distribution of the 84 most abundant families between samples, comprising 93.3–99.4% of family-assigned communities. The most abundant families found in the rhizosphere of *D. antarctica* were *Chitinophagaceae* (12.5±8.7% in samples from Galindez Island and 13.5±5.4% in samples from Anvers Island), *Sphingomonadaceae* (6.4±7.1 and 1.4±1.7%), *Comamonadaceae* (5.7±3.0 and 10.3±11.6%), *Xanthomonadaceae* (8.3±9.8 and 5.3±4.1%), *Moraxellaceae* (9.2±19.6 and 3.2±5.2%), and *Cytophagaceae* (8.2±5.8 and 2.2±1.8%). Although the abundance of most of these families differed between the two islands, the small overall sample sizes and heterogeneity between samples meant that no significant differences were detected (Table S3). The family-level composition of the *D. cespitosa* rhizosphere microbiome was similar to that of *D. antarctica*. The single dominant family in the rhizosphere microbiome of *C. quitensis* was *Micrococcaceae* (93.4%).

### Taxonomic associations with soil chemical parameters

We investigated whether soil parameters showed any significant relationship with the occurrence of specific taxa at the family level using a generalized linear model with a negative binomial distribution. No correlations were observed between prokaryotic families and the concentration of organic carbon, the С:N ratio, pH, total nitrogen, or P_2_O_5_. The content of Cd (mg kg^–1^ soil) had the strongest impact on prokaryotic communities; it correlated with *Marinicellaceae* (q=0.006), *Anaeroplasmataceae* (q=3.05×10^–5^), family JTB38 belonging to *Deltaproteobacteria* (q=0.006), family PAUC26f belonging to *Acidobacteria* (q=0.006), *Leptospiraceae* (q=0.03), *Anaerolinaceae* (q=0.03), and *Cerasicoccaceae* (q=0.04). Spearman’s rank correlation test confirmed that the abundance of these taxa negatively correlated with the content of Cd (Table S4). However, these families accounted for <0.05% of family-assigned communities, and variations did not affect the overall heterogeneity of these communities. *Moraxellaceae* (q=0.009) and *Carnobacteriaceae* (q=0.009) were positively associated with K_2_O (Table S4).

### Effects of microclimatic conditions on the composition of microbial communities

Average soil surface temperatures in populations of *D. antarctica* in December 2017, January 2018, and February 2018 are shown in Table 4. Average temperature varied between populations from 1.3 to 6.8°C in December, from 3.2 to 6.0°C in January, and from 2.9 to 4.2°C in February. Population D6 had the lowest average temperature in the three months, which appeared to be due to snow being present in the depression containing this population, and may be responsible for the lower microbial diversity associated with this population.

Temperature variations may have had different impacts on rhizosphere microbial communities in different months. The reduction in temperature differences recorded over the summer season may have affected temperature-sensitive bacteria in the communities.

The results of the Mantel test confirmed that *Actinobacteria*, *Bacteroidetes*, and *Proteobacteria* were sensitive to soil temperature changes during the three summer months (Table 5). Sensitivity to temperature changes decreased in the order of *Actinobacteria* > *Bacteroidetes* > *Proteobacteria*. The effects of temperature variations on temperature-sensitive bacterial taxa were the most apparent in December, followed by a decrease in January and a slight increase in February. Supplementary Fig. S3 shows the dependence of spatial pairwise distances matrices estimated for *Actinobacteria*.

**Fig. 5.**
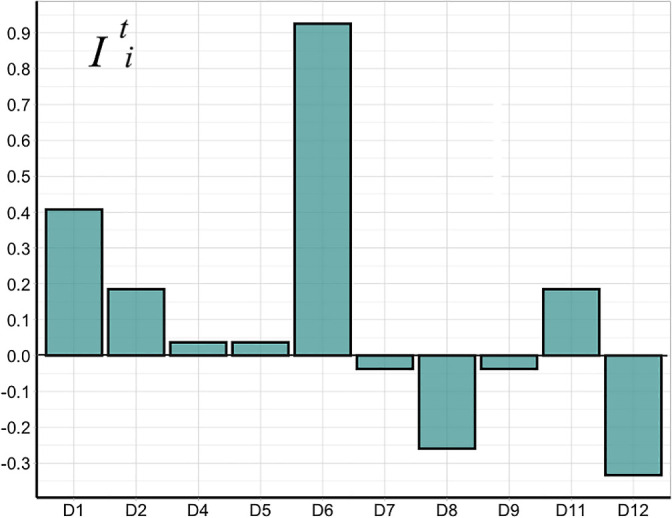
- United Soil Surface Temperature Influence Index (UTII, *I^t^_i_*) for distance matrices between *Deschampsia antarctica* rhizosphere bacterial communities.

The United Soil Surface Temperature Influence Index (UTII, *I^t^_i_*) is shown in Fig. 5, and the Soil Surface Temperature Index calculated for each month in Table S5. UTII, *I^t^_i_* was calculated to estimate the effects of temperature variations on the compositions of rhizosphere microbial communities. The probability of microbial communities matching into a ‘positive’ or ‘negative’ group formed after an extreme grouping procedure is the basis for the UTII, *I^t^_i_* calculation. UTII-positive microbial communities had more matches into the ‘positive’ group than into the ‘negative’ group. Microbial communities with a negative UTII value included two groups: a small group whose microbial communities were not influenced by temperature changes, and a larger group that were influenced by differences in other variables. UTII values of approximately zero ​​indicated that microbial groups had approximately the same matching number, which belonged to the ‘positive’ or ‘negative’ group in different months of the season.

The value of UTII, *I^t^_i_* is a function of the community composition and taxa ratio. Positive values for UTII, *I^t^_i_* in microbial communities from the populations D1, D2, D4, D5, and D11 may be due to the relatively high quantities of *Proteobacteria* and *Bacteroidetes*, which are positively influenced by temperature variations (as shown in the Mantel test). The microbial community in population D6, with the highest value for UTII, *I^t^_i_*, was dominated by *Actinobacteria*.

### Functional predictions of the metagenome

Overall, 7,364 KO profiles, 2,273 EC profiles, and 427 MetaCyc pathways were predicted in the rhizosphere microbial communities of the three plant species (Table 3). We constructed a Bray Curtis dissimilarity matrix of MetaCyc pathway abundance across samples. PCoA revealed that communities from the rhizosphere of *D. antarctica* were more similar in terms of their metabolic pathway abundance (Fig. 2B) than indicated by their ASV abundance (Fig. 2A); therefore, although these communities comprised distinct ASVs, they appeared to harbor similar functional capabilities. Microbial communities from the rhizospheres of *D. antarctica* growing on Galindez and Anvers Islands and from southern temperate *D. caespitosa* also generally had similar functional repertoires (Fig. 2B).

**Fig. 6.**
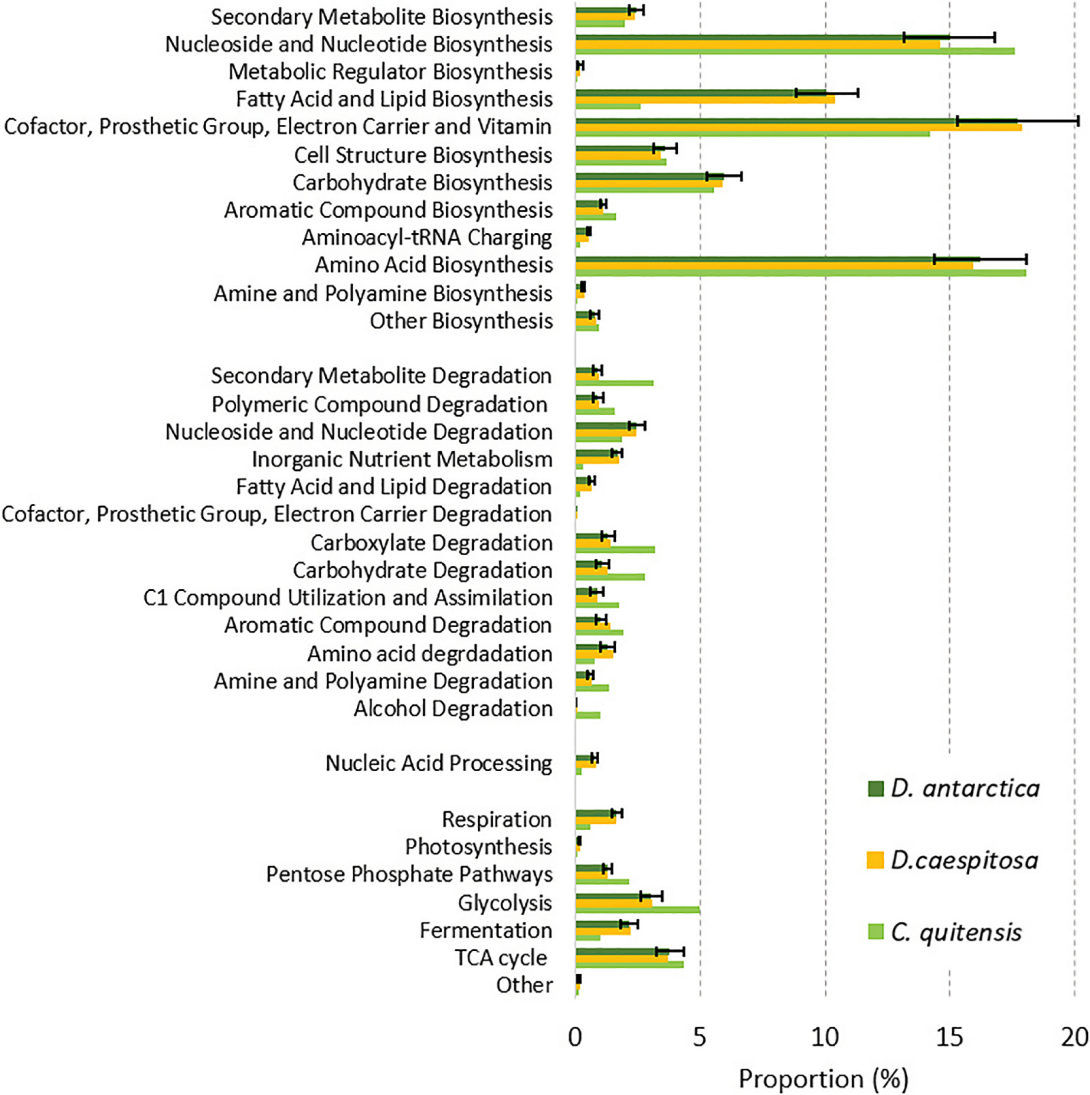
Relative abundance of different metabolic functions (MetaCyc ontology predictions) in rhizosphere metagenomes, estimated using PICRUSt 2.0.

Fig. 6 shows that the large majority of the predicted functional capacities were devoted to biosynthetic processes (overall 73.9±9.3% in *D. antarctica*, 73.4% in* D. cespitosa*, and 66.5% in *C. quitensis*) and central metabolism (overall 12.3±1.7% in *D. antarctica*, 12.2% in *D. cespitosa*, and 13.3% in *C. quitensis*). Many of the predicted functions were responsible for the degradation of various organic substrates: carboxylate (1.3±0.2% in *D. antarctica*; 1.4% in *D. cespitosa*; 3.2% in *C. quitensis*), carbohydrate (1.1±0.2% in *D. antarctica*; 1.3% in *D. cespitosa*; 3.2% in *C. quitensis*), amines and polyamines (0.6±0.1% in *D. antarctica*; 0.6% in *D. cespitosa*; 1.3% in *C. quitensis*), polymers (0.9±0.2% in *D. antarctica*; 0.9% in *D. cespitosa*; 1.5% in *C. quitensis*), and aromatic compounds (1.0±0.2% in *D. antarctica*; 1.3% in *D. cespitosa*; 1.9% in *C. quitensis*).

**Fig. 7.**
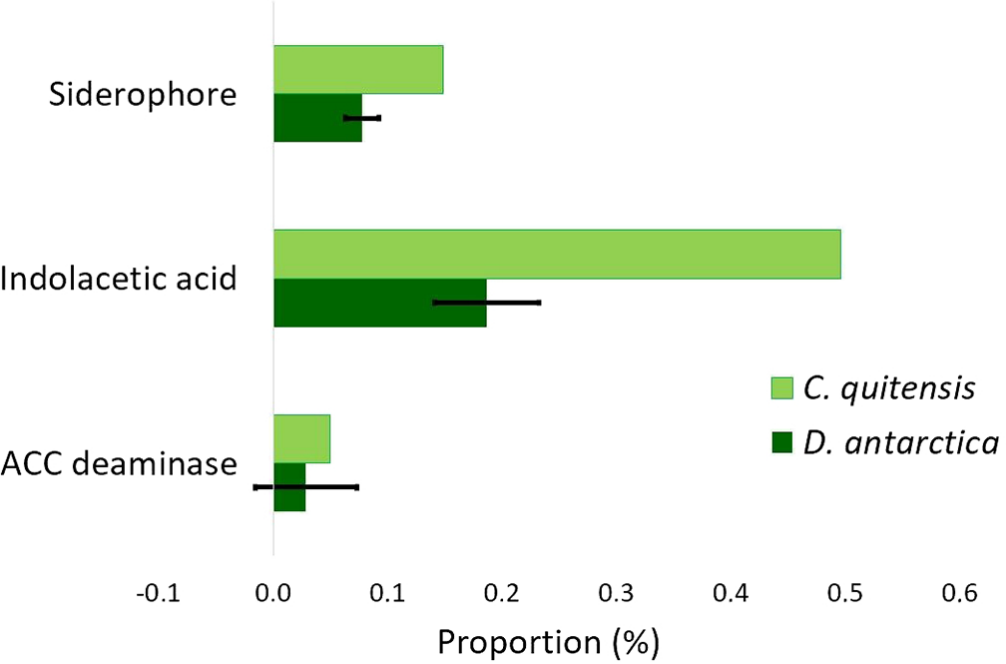
Relative abundance of KEGG orthologs involved in the synthesis of siderophores, IAA, and ACC deaminase

The presence of functions related to secondary metabolite synthesis and amino acid degradation implies that rhizosphere microbial communities may be involved in the synthesis of siderophores, ACC deaminase, or phytohormones like IAA. We estimated the proportion of KEGG orthologs involved in the synthesis of these compounds using the MetaCyc and KEGG Orthologs databases (Table S6). These orthologs accounted for up to 0.5% of the predicted orthologs (Fig. 7).

## Discussion

The present study investigated the diversity and functional repertoire of the microbiomes of the rhizosphere soil of vascular plants growing in two locations in the central maritime Antarctic, Galindez and Anvers Islands. Carbon and nitrogen contents markedly differed in soils from these two locations. Rhizosphere soil from Galindez Island was rich in nutrients derived largely from ornithogenic sources. In contrast, the carbon content in soil from Anvers Island was very low due to more recent exposure from the glacier retreat in the 20^th^ Century and the associated shorter period of pedogenesis (Strauss *et al.*, 2009). Deglaciation on Galindez Island began markedly earlier, but not later than 2300–1200 cal B.P. (Yu *et al.*, 2016).

We expected significant differences in diversity and community compositions between rhizosphere microbiomes from plants growing on Galindez and Anvers Islands. However, the rhizosphere microbiomes of *D. antarctica* from both locations were equally diverse, with high Shannon index values and numbers of ASVs. Different nutrient contents and periods of pedogenesis did not influence the diversity of these microbial communities. Differences in nutrient contents have been suggested to affect the microbial biomass in soil (Horwath, 2017), and higher estimates of the microbial biomass in rhizosphere soil on Galindez Island are expected. A markedly less diverse microbiome was detected in the rhizosphere of the second Antarctic native plant, *C. quitensis*, with the caveat that only a single sampling site was available for this species. The plant growth stage is another important factor potentially affecting the rhizosphere microbial community; however, it did not appear to be a driving factor in this case because the plants considered in the present study were in a similar growth stage. Due to the short summer season in Antarctica, plants start vegetative growth as soon as the snow melts which, given the proximity of the different population sampling points, may have been at similar times in the season.

Microscale variations in environmental factors may influence the compositions of the rhizosphere microbiomes of *D. antarctica*, which showed high ASV heterogeneity between the sampled communities as well as high number of unique ASVs in each sample. Diversity indexes obtained in the present study indicated that the diversities of the assessed rhizosphere microbiomes were very similar to those of Antarctic vascular plants growing on King George Island in the South Shetland Islands (Teixeira *et al.*, 2010), 400‍ ‍km north of Galindez and Anvers Islands. The microbiome of *D. cespitosa*, growing north of the Drake Passage in Chilean Patagonia, also had very similar α-diversity values. The present results are consistent with previous findings reported by Yergeau *et al.* (2007), where the absence of a latitudinal influence on bacterial diversity was noted in moss-covered soil across the maritime Antarctic, unlike in bare soils. The rhizosphere may provide a favorable habitat for bacteria, with vegetation shielding underlying soil from environmental extremes (Harris and Tibbles, 1997; Yergeau *et al.*, 2007).

Rhizosphere microbiomes included high proportions of unique ASVs and intrinsic heterogeneity. The majority of ASVs identified were affiliated to the phyla *Proteobacteria*, *Bacteroidetes*, *Actinobacteria*, *Acidobacteria*, and *Verrucomicrobia*. These bacterial phyla are recognized as core soil phyla in a wide variety of soils (Delgado-Baquerizo *et al.*, 2018), including those in the Antarctic (Yergeau *et al.*, 2007; Chong *et al.*, 2012).

The phylum-level composition of microbiomes from Galindez and Anvers Islands showed few differences, except for the slightly higher proportions of *Acidobacteria* and *Verrucomicrobia* in samples from Anvers Island. *Acidobacteria* are generally considered to be oligotrophs that are capable of metabolizing recalcitrant carbon sources, and are expected to colonize nutrient-poor substrates prior to copiotrophic taxa (Fierer *et al.*, 2007; Ward *et al.*, 2009; Yergeau *et al.*, 2012). Some members of *Verrucomicrobia* are also oligotrophs (Shen *et al.*, 2017) and are capable of hydrolyzing polysaccharide substrates (Cardman *et al.*, 2014). The proportions of the other major phyla (*Bacteroidetes*, *Actinobacteria*, *Proteobacteria*), the members of which are typically associated with higher carbon content soils (Fierer *et al.*, 2007), did not significantly differ between the nutrient-rich soil of Galindez Island and nutrient-poor soil of Anvers Island.

The microbial community present around the root system of *D. cespitosa* generally had a similar composition to that of *D. antarctica*. Plant species affect the composition of the rhizosphere soil microbiota by secreting root exudates (Hartmann *et al.*, 2009; Cesco *et al.*, 2010; Bulgarelli *et al.*, 2013; Hu *et al.*, 2018), which may be a reason for the pattern observed.

Some of the results of the present study were not consistent with the findings reported by Teixeira *et al.* (2010) who conducted a rhizosphere study in the South Shetland Islands. They identified *Firmicutes* as the most abundant phylum and rarely found *Acidobacteria*. Methodological differences in microbiome sampling may underlie this discrepancy. In the present study, soil that aggregated around plant roots was sampled, while sampling by Teixeira *et al.* (2010) included washing the roots in order to include root-adherent cells. This may have affected the microbial diversity detected because distinct communities may populate the root surface and root-aggregated soil (Mwajita *et al.*, 2013).

Our rhizosphere microbiome data for *D. antarctica* are similar to those from the rhizosphere microbiomes of *C. quitensis* and mixed populations of *D. antarctica* and *C. quitensis* growing on Livingston Island in the South Shetland Islands (Molina-Montenegro *et al.*, 2019), with both studies using similar sampling procedures. The same phyla were also found in the rhizospheres of the two flowering plants and bryophytes growing on King George Island (Roesch *et al.*, 2012). The rhizosphere microbiome of the mixed *D. antarctica* and *C. quitensis* community from Livingston Island had more *Acidobacteria* and *Actinobacteria* than that from Galindez Island (approximately 10% vs. 4% of *Acidobacteria*, and 25% vs. 9% of *Actinobacteria*). Similarly, soils from King George Island had higher estimates of *Acidobacteria* (17.7%) than our data (Roesch *et al.*, 2012). The rhizosphere microbiome of *C. quitensis* from Galindez Island assessed in the present study contrasted with that of *D. antarctica* and also with previous studies, identifying *Actinobacteria* as the dominant phylum (93%). Although it is easy to conclude that the plant species itself drives the difference in the microbiome composition, findings by Molina-Montenegro *et al.* (2019) showed that the rhizosphere of *C. quitensis* from Livingston Island included the typical soil microbiota.

Total carbon, nitrogen, and moisture were identified as key environmental factors influencing the soil bacteria community on Livingston Island (Ganzert *et al.*, 2011). Stomeo *et al.* (2012) reported that K, C, Ca, and moisture affected the composition of microbial communities from Miers Valley (a coastal valley in the McMurdo Dry Valleys, continental Antarctica). pH was the best predictor of the microbiome composition of pristine soil as well as soil impacted by penguins, seals, and humans in the Fildes Peninsula, followed by PO_3_-P, organic C, and organic N (Wang *et al.*, 2015). Similarly, pH played a pivotal role in the ecology of bacteria in soils on Signy Island (South Orkney Islands) (Chong *et al.*, 2009; 2010) and arid sites on Alexander Island and islands in Ryder Bay off the west coast of the Antarctic Peninsula (Chong *et al.*, 2012). Conductivity, moisture, and copper, lead, and nickel contents were also limiting factors for microbial communities in the soils of Signy Island (Chong *et al.*, 2010). A previous study indicated that the soil microbial biomass was highly sensitive to elevated UV-B radiation and CO_2_ concentrations, and also suggested that UV-B altered the amount of N held within the soil microbial biomass (Johnson *et al.*, 2002). Carbon, nitrogen and phosphorus contents differed in soils from Galindez and Anvers Islands; however, no relationships were observed between bacterial taxa and these nutrients in the present study. Furthermore, a taxa association was not found with pH. The relatively rare families *Moraxellaceae* and *Carnobacteriaceae* were associated with the content of K_2_O; however, this does not explain the overall dissimilarity between microbial communities. Microclimatic conditions may have affected rhizosphere microbial communities because the temperature of the soil surface varied by several degrees between the different sampling locations. The low diversity of microbial communities in sampling site D6 may be related to it having the lowest temperature among the sites tested. The Mantel test confirmed that bacteria belonging to *Actinobacteria*, *Bacteroidetes*, and *Proteobacteria* were sensitive to temperature fluctuations, and variations between most of the microbial communities assessed on Galindez Island were associated with temperature variations according to UTII values. Other factors not examined in the present study, such as freeze-thaw cycles (Lim *et al.*, 2020) and moisture (Stomeo *et al.*, 2012), may also have contributed to the variations observed in the microbiome structure.

PICRUSt2.0 was applied to predict the microbial community functional potential; however, this tool has a number of limitations and the interpretation of results is constrained by the availability of sequenced reference genomes (Douglas, G., *et al.* 2019 PICRUSt2: An improved and customizable approach for metagenome inference. biorxiv. https://doi.org/10.1101/672295). A large proportion of the unclassified 16S rRNA gene sequences detected at the family and genus levels in the present study will reduce the accuracy of predictions, and the predictions made here were considered to be the most relevant to the identified portion of the microbial communities. With that caveat, predictions of microbial functions in the rhizosphere using PICRUSt2.0 revealed that in contrast to the strong heterogeneity in community compositions and abundance, these microbiomes were more similar in functional terms as indicated by the metabolic MetaCyc pathways. Environmental conditions, such as exposure to freeze-thaw cycles, low temperatures, and the availability of plant derivatives, may exert selective pressure on microbiomes inhabiting the rhizosphere and promote the selection of taxa and communities with similar functional repertoires. The majority of functions identified were related to bacterial biosynthetic processes and central metabolism. Molina-Montenegro *et al.* (2019) suggested that particular functional traits were selected for the rhizosphere of Antarctic plants. They reported that “replication, recombination and repair” was the most abundant eggNOG gene category in the communities present in the rhizosphere of Antarctic vascular plants on Livingston Island. These functions include energy production, nutrient transportation, cell membrane functions, and tolerance to abiotic stress (Barrientos-Díaz *et al.*, 2008). A PICRUSt ana­lysis of cultured bacteria from the rhizosphere soil of *D. antarctica* and *C. quitensis* from King George Island (da Silva *et al.*, 2017) identified a large number of genes involved in metabolism (approximately 1,700 different genes per ASV), suggesting the high metabolic capacities of these isolates.

The present results indicate that, in addition to servicing central metabolic processes, rhizosphere microbial communities are involved in primary soil development and may also be beneficial for the growth of Antarctic vascular plants. A high proportion of metabolic pathways were predicted that are responsible for the degradation of organic substrates, including polymers and aromatic compounds, particularly plant-derived substrates such as lignin and lignin-derived aromatic compounds. The MetaCyc pathways P184-PWY, PWY-6338, PWY-6339, PWY-7097, and PWY-7098, which are involved in the degradation of lignin derivatives, including protocatechuate (Hara *et al.*, 2000), vanillin and vallinate (Gold and Alic, 1993; Chen *et al.*, 2012), and syringate (Masai *et al.*, 2007), were detected among the predicted functions. These predictions are consistent with taxonomic data, where the chemoheterotrophic bacterial family *Chitinophagaceae* was dominant in all communities. This group includes diverse aerobic and anaerobic chemoheterotrophic bacteria that degrade chitin, lignin, and cellulose (del Rio *et al.*, 2010; Chung *et al.*, 2012). *Xanthomonadaceae*, *Moraxellaceae*, *Cytophagaceae*, *Comamonadaceae*, and *Sphingomonadaceae*, which are capable of degrading macromolecules (Abaydulla *et al.*, 2012; Xu *et al.*, 2014), were the other dominant bacterial families. These bacteria were abundant in both carbon-rich soil from Galindez Island and carbon poor-soil from Anvers Island. On both islands, the close proximity of plant root systems may contribute to the selection of bacteria that metabolize plant derivatives, even though the more general accumulation of labile carbon in the soils of Galindez Island was promoted by the markedly longer period of soil development.

Rhizosphere microorganisms facilitate plant growth by producing plant growth-promoting compounds, acquiring nutrients, and preventing microbial invasion (Lugtenberg and Kamilova, 2009; Glick, 2012). Our ana­lyses revealed that the abundance of orthologs involved in the synthesis of IAA, siderophores, and ACC deaminase reached up to 0.5% of the predicted orthologs. However, PICRUST 2.0 results revealing plant-growth promoting traits need to be validated using metagenomic and metatranscriptomic data.

## Conclusions

The rhizosphere microbiomes of *D. antarctica* from two distant regions in the central maritime Antarctic had similar diversity and taxonomic compositions even though the soil from these regions had markedly different nutrient contents and periods of pedogenesis. In comparison with the comparatively well described region of the South Shetland Islands in the northern maritime Antarctic, there was no suggestion of reduced diversity in rhizosphere microbiomes, which is consistent with the findings of previous studies on the microbiomes of moss-covered soil in the Antarctic Peninsula and Scotia Arc. Vegetation cover may protect the underlying soil and the rhizosphere microbiome from exposure to environmental extremes. Variations in environmental parameters at the microscale level may still occur within these habitats, underlying the overall heterogeneity observed between microbial communities; however, few significant relationships were detected in the present study. These parameters include the physical and chemical characteristics of soil, temperature fluctuations, water availability, and freeze-thaw cycles.

Rhizosphere microbial communities were similar in terms of their potential functional capacity, as revealed by the PICRUSt2.0 tool. Besides basic metabolic processes (biosynthesis and central metabolism), a large component of their predicted functional repertoire was devoted to breaking down organic compounds and their derivatives, supporting the involvement of the rhizosphere microbiota in the development of primary Antarctic soil and the accumulation of labile carbon. Bacterial functions responsible for plant growth promotion potentially facilitate plant resource acquisition and modulate phytohormone levels. Therefore, these mechanisms may be a significant factor in Antarctic vascular plant performance.

## Funding

This work was supported by the National Antarctic Scientific Center of Ukraine (State Target Scientific and Technical Program for Antarctic Research in 2011–2020), the State Special-Purpose Research Program in Antarctica for 2011–2023. Peter Convey is supported by NERC core funding to the BAS ‘Biodiversity, Evolution and Adaptation’ Team.

## Citation

Prekrasna, I., Pavlovska, M., Miryuta, N., Dzhulai, A., Dykyi, E., Convey, P., et al. (2022) Antarctic Hairgrass Rhizosphere Microbiomes: Microscale Effects Shape Diversity, Structure, and Function. *Microbes Environ ***37**: ME21069.

https://doi.org/10.1264/jsme2.ME21069

## Supplementary Material

Supplementary Material

## Figures and Tables

**Table 1.** Description and coordinates of sampling locations; IVC-individual vegetation cover of *Deschampsia* sp.

Sample ID	Area	Locations, description, coordinates (Latitude, Longitude)	Type of Vegetation	IVC*, %
PA0	Anvers	Gamage Point; near Terra lab, gravel; 22 m a.s.l.; -‍64.77455, -‍64.051617	*D. antarctica* on gravel with *Psoroma* sp.	<1
PA20		Gamage Point; coastal rock near the fuel pump house, gravel, 10 m a.s.l.; -‍64.774767, -‍64.053267	*D. antarctica* with bryophytes	1–3
PA26		Gamage Point; coastal rock near Survey Control Monument, gravel, 17 m a.s.l; -‍64.774183, -‍64.055367	*D. antarctica* with bryophytes	1
D1	Galindez	Meteo Point; rocky coast of Marina Point, gravel, 13‍ ‍m‍ ‍a.s.l; -‍65.244767, -‍64.2558	*D. antarctica* affected by penguin nitrate input with bryophytes and *Prasiola crispa*	1
D2		Marina Point; near the main station building; gravel, 12‍ ‍m‍ ‍a.s.l; -‍65.245667, -‍64.256817	*D. antarctica* with bryophytes	30
D4		Penguin Point; rock tower, limited guano input; gravel, 7‍ ‍m‍ ‍a.s.l.; -‍65.248600, -‍64.238230	*D. antarctica* with *P. crispa* on limpet deposits	3
D5		Hovorukha Dome top under Anna Hill; gravel, 45‍ ‍m‍ ‍a.s.l; -‍65.248267, -‍64.245433	*D. antarctica* with *bryophytes*	1
D6		Roztochia Rigde; rock surface, gravel, 19‍ ‍m‍ ‍a.s.l.; -‍65.248100, -‍64.243240	*D. antarctica* and *C. quitensis* with bryophytes	3
D7		Krapla Rock; coastal rock, gravel, 16‍ ‍m‍ ‍a.s.l.; -‍65.247017, -‍64.243167;	*D. antarctica* on limpet deposits	5
D8		Marina Point; near the diesel station, gravel; 3‍ ‍m‍ ‍a.s.l.; -‍65.24585, -‍64.25765	*D. antarctica* with bryophytes	3
D9		Neck Ridge; coastal rocks, gravel, 14‍ ‍m‍ ‍a.s.l.; -‍65.245467, -‍64.249867	*D. antarctica* with bryophytes	15
D11		Cemetry Ridge; rock surface, limpets, gravel, 17‍ ‍m‍ ‍a.s.l.; -‍65.246317, -‍64.248533	*D. antarctica* with bryophytes	1
D12		Stella Point; coastal rock, gravel and limpet shells, 10‍ ‍m‍ ‍a.s.l.; -‍65.24745, -‍64.252733	*D. antarctica* with bryophytes	10
PuA	Punta Arenas	Shore of the Magellan Strait; alluvium, 1 m a.s.l.; -‍53.163967, -‍70.896633	in a coastal sandy community with *Leymus arenarius* (L.) Hochst. and *Matricaria sp*., *Rumex sp.* near a coastal stream	6

**Table 2.** Chemical parameters of rhizosphere soil from *Deschampsia antarctica* and *Colobanthus quitensis* populations*

Sample ID	Type of soil	C org, %	C:N	pH H_2_0	Total content, %				Trace elements, mg kg^–1^						
					N	P_2_O_5_	K_2_O		Cu	Zn	Pb	Cd	Ni	Mn	Fe
D1*	Leptosol	47	18.1	5	2.6	1.7	0.2		43.9	50.8	63.8	0.7	1.6	54.3	13,200
D2*	Leptosol	27	10.5	6.3	2.6	1.9	0.1		192.5	616.5	1,760	29.8	11.4	252.5	15,700
D4*	Ornithic Leptosol	10.6	8.9	7.6	1.2	8.3	0.2		0.2	0.4	5.8	0.6	2.2	0.6	4.5
D5*	Ornithic Leptosol	21.3	10.5	7.4	2.0	7.1	0.2		0.3	0.5	5.5	1	2.5	1	5.5
D6*	Leptosol	41.9	21.1	6.7	2.0	3.2	0.2		0.2	8	4.8	10.1	4.4	36.2	3.8
D7	Leptosol	nd	nd	nd	nd	nd	nd		nd	nd	nd	nd	nd	nd	nd
D8*	Gleysol	41.2	14	5.2	2.9	2.8	0.3		70	52.2	12.4	2	2.2	90	12,200
D9*	Leptosol	32.8	21.4	6.8	1.5	1.0	0.2		1,856	667.1	741.3	20.9	12.2	77.1	18,600
D11*	Leptosol	45.1	18	6.2	2.5	2.0	0.5		18	55.4	6.3	2.8	1.5	104.3	14,900
D12*	Leptosol	63	22.8	5.5	2.8	1.6	0.2		30.5	53.2	6.9	2.8	3	76.3	25.8
PA0	Leptosol	0.3	17.6	6.5	0.02	3.5	0.3		123	93	18.7	3.7	6.5	267.5	24,500
PA20	Leptosol	0.2	18.5	6.1	0.01	4.0	0.3		157	139	38	5.4	7.4	286.5	26,900
PA26	Leptosol	1.0	7.8	6.5	0.1	3.0	0.4		164	250	14.3	12.2	6.1	339	20,800

nd—no data available * Data from Parnikoza *et al.*, 2016

**Table 3.** Estimated ASV richness and diversity indices for 16S rRNA libraries of rhizosphere samples from *Deschampsia antarctica*, *Deschampsia cespitosa*, and *Colobanthus quitensis*

Sample ID	No. of reads	No. of ASVs	Unique ASVs, %	Shannon Index	No. of EC numbers	No. of KEGG Orthologs	No. of MetaCyc pathways
D1	75,851	1,392	25.2	9.4	1,067	2,007	404
D2	77,380	1,474	53.8	9.2	1,027	2,034	402
D4	76,704	1,501	30.1	9.5	1,002	1,952	412
D5	92,998	1,035	46.7	8.5	1,067	2,054	406
D6	92,778	125	54	1.0	777	1,430	370
D7	107,201	582	19.6	5.2	1,001	1,909	385
D8	106,125	765	59.0	7.2	1,035	1,995	389
D9	101,857	955	26.4	7.7	1,049	2,064	380
D11	94,864	953	22.6	7.8	1,076	2,087	395
D12	99,431	1,070	61.2	8.0	1,006	1,900	395
PA0	102,248	597	41.2	7.0	1,013	1,991	389
PA20	79,680	1,228	63.3	8.9	1,014	1,910	404
PA26	92,449	1,228	56.7	8.9	1,030	1,967	395
PuA	78,018	1,113	24.5	8.8	1,040	2,053	396
**Total**	**1,184,806**	**8,298**			**2,273**	**7,364**	**427**

**Table 4.** Average soil surface temperatures in December 2017, January 2018, and February 2018 for each *Deschampsia antarctica* population studied on Galindez Island. The mean±standard deviation/variance are shown.

Population	Average T°C, 12.2017	Range of T°C, 12.2017	Average T°C, 01.2018	Range of T°C, 01.2018	Average T°C, 02.2018	Range of T°C, 02.2018
D1	5.7±2.7/7.5	0.3–10.5	5.4±2.1/4.6	1.3–9.0	4.1±1.6/2.7	1.7–7.4
D2	4.1±2.5/6.1	0.2–8.5	4.5±2.0/4.1	0.1–8.2	4.2±1.8/3.4	1.2–7.8
D4	4.9±2.7/7.5	–0.2–9.7	4.3±2.1/4.3	0.1–8.2	3.6±1.8/3.1	0.9–9.1
D5	6.8±3.4/11.6	0.7–14.0	6.0±3.1/10.1	0.1–12.5	3.3±1.4/2.1	0–5.9
D6	1.3±2.0/4.0	–0.1–7.0	3.2±1.7/2.8	0–6.2	2.9±1.1/1.3	1.0–5.7
D7	5.1±2.8/8.0	0.3–11.5	4.5±2.0/4.1	0.1–8.1	3.5±1.5/2.2	1.4–7.5
D8	3.7±1.4/2.0	1.5–6.3	4.3±1.1/1.1	1.9–6.4	3.9±1.0/1.0	2.1–5.8
D9	5.3±2.7/7.1	0.1–9.6	4.8±2.2/4.8	0.1–9.0	4.0±1.5/2.3	1.7–6.8
D11	5.8±2.9/8.3	0.1–11.0	5.2±2.3/5.3	0.1–9.0	4.2±1.6/2.7	1.7–7.2
D12	5.0±2.5/6.4	0.2–9.1	4.6±2.1/4.6	0.1–8.7	3.9±1.5/2.7	1.7–7.2

**Table 5.** Influence of soil surface temperature on the relative abundance of bacterial phyla estimated by the Mantel test. Test values were calculated using a regression technique*.

Phylum	ΔT1**	ΔT2**	ΔT3**
*Acidobacteria*	0.817	0.000	0.559
*Actinobacteria*	76.798	24.510	28.036
*Bacteroidetes*	41.409	15.609	18.103
*Chlorobi*	1.892	1.935	0.516
*Chloroflexi*	0.473	1.075	0.000
*Crenarchaeota*	1.677	1.505	0.602
*Cyanobacteria*	0.086	0.860	0.086
*Firmicutes*	1.505	1.720	0.731
*Gemmatimonadetes*	1.247	1.720	0.387
*Proteobacteria*	18.017	6.966	7.310
*Verrucomicrobia*	0.860	0.000	1.806
*Planctomycetes*	0.473	0.602	0.000

Notes: * test value F_1,n–2_=t^2^_n–2_=(n–2)R^2^/(1–R^2^), *n*=45, for the upper limit 5% (α=0.05) of the F-distribution point value is 4.08 for *n*=45 **ΔT1—December 2017, ΔT2—January 2018, ΔT3—February 2018
