# Supplementary material for: Antarctic Hairgrass Rhizosphere Microbiomes: Microscale Effects Shape Diversity, Structure, and Function
Source: Microbes Environ. 2022 Jun 15;37(2):ME21069. doi: 10.1264/jsme2.ME21069 (PMC9530728; doi:10.1264/jsme2.ME21069)
Supplement: Supplementary file 1 — Supplementary Material [file 37_21069_s1.pdf]

Supplementary data

Antarctic hairgrass rhizosphere microbiomes: microscale effects shape diversity, structure and function

Ievgeniia Prekrasna[1], Mariia Pavlovska[1,7], Natalia Miryuta[1], Artem Dzhulai[1], Evgen Dykyi[1], Peter Convey[2,3], Iryna Kozeretska[1], Tymur Bedernichek[4], Ivan Parnikoza*[1,5,6]

[1]State Institution National Antarctic Scientific Center, 16 Taras Shevchenko Blvd., 01601, Kyiv, Ukraine
[2]British Antarctic Survey, NERC, High Cross, Madingley Road, Cambridge, CB3 0ET, UK
[3]Department of Zoology, University of Johannesburg, PO Box 524, Auckland Park 2006, South Africa
[4]M.M. Gryshko National Botanical Garden, 1 Timiryazev Str., 01014, Kyiv, Ukraine
[5]Institute of Molecular Biology and Genetics, 150 Zabolotnogo Str., 03143, Kyiv, Ukraine
[6]National University of “Kyiv-Mohyla Academy”, st. Skovorody, 2, Kyiv, 04070, Ukraine
[7]National University of Life and Environmental Sciences of Ukraine, 15 Heroiv Oborony Str., 03041, Kyiv, Ukraine

Corresponding author: Ivan Parnikoza, email: ivan.parnikoza@uac.gov.ua

State Institution National Antarctic Scientific Center, Kyiv, Ukraine, Department of Biology and Ecology, 01601, Taras Shevchenko Boulevard, Kyiv, Ukraine
Phone: +38 **044 246 38 80**

Figures

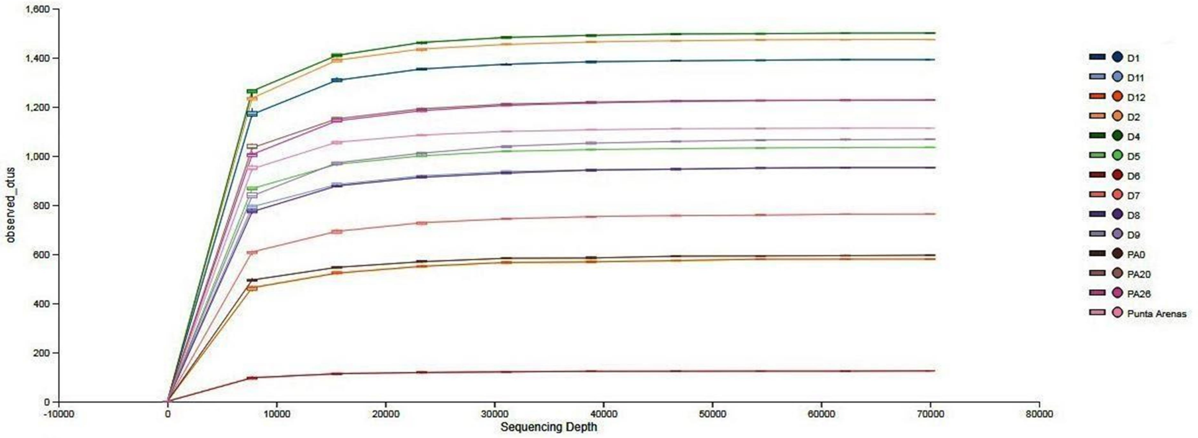

Figure S1. Rarefaction curves of partial sequences of bacterial 16S rRNA genes from the rhizosphere of vascular plants: D1, D2, D4, D5, D7, D8, D9, D11 - samples *of D. antarctica rhizosphere,* Galindez Island; PA0, PA20, PA26 - samples *of D. antarctica* rhizosphere, Anvers Island; PuA – sample of *D. caespitosa* rhizosphere, Punta Arenas; D6 - sample of *C. quitensis* rhizosphere, Galindez Island

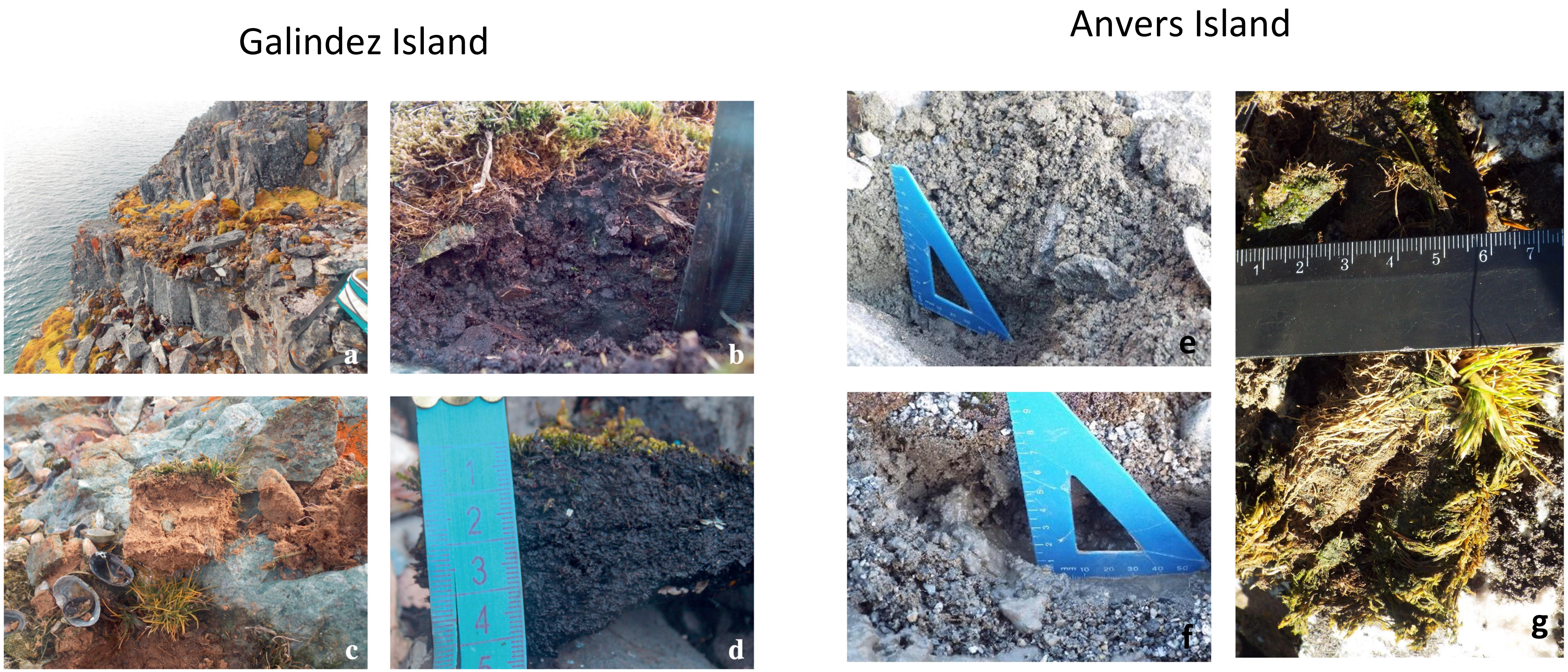

Figure S2 – Selected sampling sites and soils on Galindez (a-d) and Anvers (e – g) Islands. a and b: sample location D9; c: sample location D4 at Penguin Point; d: sample location D2; e – sample location PA20; f – sample location PA0; g – sample location PA26.

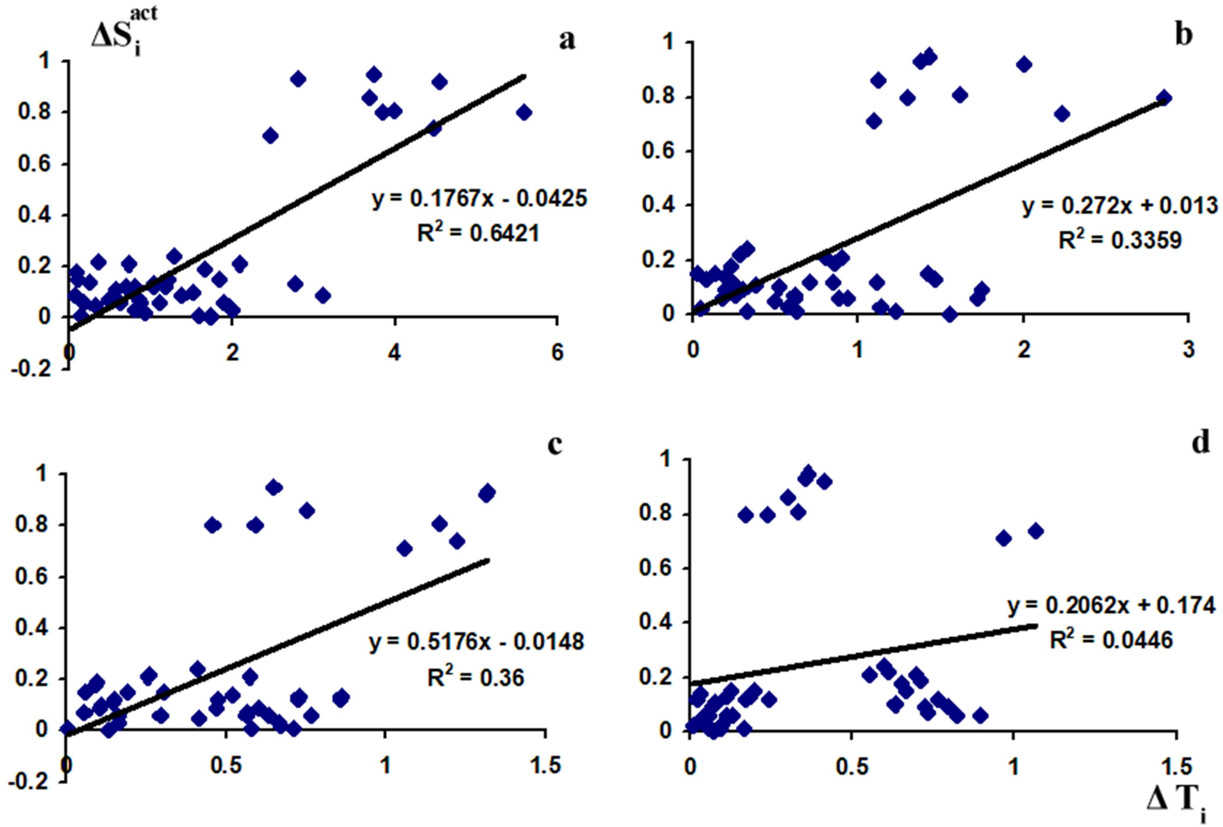

Figure S3. Dependence of spatial pairwise distances set of the *Actinobacteria*'s relative abundance on temperature pairwise spatial distances set in the Galindez Island in the 2017/18 season: a - December 2017, b - January 2018, c - February 2018, d - March 2018. ΔT_i - temperature pairwise spatial distances set, ΔS^{act}_i - pairwise spatial distances set of the *Actinobacteria's* relative abundance.

Tables

Table S1 – Ubiquitous OTUs assigned to the lowest taxonomic level and their relative abundance in the samples

Taxonomic assignment	Relative abundance in samples, %													
	D1	D2	D4	D5	D6	D7	D8	D9	D11	D12	PA0	PA20	PA26	PuA
Arthrobacter sp.	1.58	0.17	0.31	0.87	88.48	1.83	0.35	0.26	0.33	0.38	0.69	0.70	0.71	0.67
Psychrobacter sp.	0.08	0.09	0.06	0.06	0.07	0.06	0.11	0.07	12.81	34.57	0.11	0.05	0.09	0.20
Staphylococcus equorum	0.03	0.03	0.02	0.03	0.03	0.04	0.02	0.03	0.04	18.05	0.04	0.02	0.02	0.04
Myxococcales	1.24	0.36	0.94	0.77	0.03	1.95	0.43	0.11	0.04	0.04	0.03	0.47	3.09	0.08
Clostridium bowmanii	0.02	0.17	0.01	0.64	0.02	0.74	0.08	0.23	0.19	0.05	0.04	1.24	1.74	0.57
Salinibacterium sp.	0.53	0.13	0.26	0.16	0.12	0.61	0.23	0.50	0.24	0.08	0.05	0.29	0.67	0.22

Table S3 – Comparison of the abundance of bacterial families in communities obtained from Galindez and Anvers Island using Kruskal-Wallis rank sum test

**Family**	**H**	**p-value**		**Family**	**H**	**p-value**
Chitinophagaceae	0.69	0.41		Isosphaeraceae	0.42	0.52
Xanthomonadaceae	0.01	0.93		Bdellovibrionaceae	0.21	0.64
Comamonadaceae	0.01	0.93		Bacillaceae	4.52	0.06
Cytophagaceae	3.09	0.08		Kineosporiaceae	1.03	0.31
Sphingomonadaceae	1.44	0.23		Pseudomonadaceae	1.03	0.31
Micrococcaceae	0.21	0.64		Erythrobacteraceae	1.92	0.17
Microbacteriaceae	0.21	0.64		Staphylococcaceae	0.42	0.52
Rhodobacteraceae	0.42	0.52		Rhizobiaceae	0.08	0.78
Hyphomicrobiaceae	0.42	0.52		Nannocystaceae	0.31	0.58
Intrasporangiaceae	1.03	0.31		Alteromonadaceae	0.21	0.64
Ellin6075	1.44	0.23		Coxiellaceae	0.08	0.78
Opitutaceae	0.21	0.64		Pseudonocardiaceae	0.04	0.85
A4b	1.92	0.17		Nostocaceae	0.70	0.40
Moraxellaceae	0.42	0.52		Frankiaceae	2.50	0.11
Haliangiaceae	0.01	0.93		Pseudanabaenaceae	0.22	0.64
Oxalobacteraceae	0.01	0.93		EB1017	0.22	0.64
Phormidiaceae	0.42	0.52		Planococcaceae	0.55	0.46
Caulobacteraceae	0.69	0.41		Deinococcaceae	0.01	0.93
Acetobacteraceae	0.08	0.78		Solibacteraceae	1.27	0.26
Sinobacteraceae	0.01	0.93		Nitrosomonadaceae	0.43	0.51
Saprospiraceae	2.19	0.14		Parachlamydiaceae	1.44	0.23
Verrucomicrobiaceae	0.69	0.41		Methylophilaceae	0.04	0.85
Bradyrhizobiaceae	0.69	0.41		Syntrophobacteraceae	0.22	0.64
Nakamurellaceae	1.44	0.23		Carnobacteriaceae	0.01	0.93
Pirellulaceae	0.55	0.46		Piscirickettsiaceae	1.05	0.31
Gemmatimonadaceae	0.08	0.78		Solirubrobacteraceae	3.20	0.07
Phyllobacteriaceae	3.09	0.08		Gaiellaceae	0.75	0.39
Clostridiaceae	1.44	0.23		Ellin5301	0.75	0.39
Rhodospirillaceae	0.21	0.64		EB1003	1.55	0.21
Polyangiaceae	1.44	0.23		Flammeovirgaceae	0.57	0.45
Gemmataceae	0.21	0.64		Nitrospiraceae	0.35	0.55
Micromonosporaceae	1.93	0.16		Acidobacteriaceae	3.52	0.06
C111	0.08	0.78		Conexibacteraceae	0.62	0.43
Cryomorphaceae	0.69	0.41		Koribacteraceae	1.80	0.18
Nocardioidaceae	0.31	0.58		Ktedonobacteraceae	0.74	0.39
Nocardiaceae	0.01	0.93		S47	0.74	0.39
Flavobacteriaceae	0.69	0.41		Aeromonadaceae	0.01	0.92
Hyphomonadaceae	1.24	0.27		Nitrososphaeraceae	0.23	0.63
OM60	1.19	0.27		Thermogemmatisporaceae	1.80	0.18

Table S4 – Association of rhizosphere bacteria at family level with chemical parameters of the soil.

Para-meter	Bacterial family (class/phylum)	logFC	logCPM	LR	P value	FDR	S	rho	P value
Cd	[Marinicellaceae] (Gammaproteobacteria/Proteo-bacteria)	-0.40	8.82	17.50	2.9e-05	0.006			
	Anaeroplasmataceae (Mollicutes/Tenericutes)	-0.40	8.84	17.40	3.1e-05	0.006	365.7	-0.66	0.03
	JTB38 (Deltaproteobacteria/ Proteobacteria)	-0.38	8.46	16.16	5.8e-05	0.006	368.7	-0.67	0.02
	PAUC26f (Adicobacteria/Acidobacteria)	-0.37	8.23	15.46	8.4e-05	0.006	368.7	-0.674	0.02
	Leptospiraceae (Spirochaetia/Spirochaetes)	-0.30	7.02	11.78	6.0e-04	0.032	368.7	-0.67	0.02
	Anaerolinaceae (Anaerolineae/Chloroflexi)	-0.35	7.96	11.77	6.0e-04	0.032	383.2	-0.74	0.01
	OM60 (Gammaproteobacteria Proteobacteria)	-0.45	10.07	10.91	9.6e-04	0.044	353.2	-0.61	0.05
	Iamiaceae (Adicobacteria/Acidobacteria)	-0.29	6.72	10.59	1.1e-03	0.045	390.8	-0.78	0.01
	[Cerasicoccaceae] (Opitutes/Verrucomicrobia)	-0.28	6.55	10.47	1.2e-03	0.045			
K_2O	Moraxellaceae (Gammaproteobacteria/ Proteobacteria)	25.65	14.80	17.81	2.4e-05	0.009	70.7	0.68	0.02
	Carnobacteriaceae (Bacillus/Firmicutes)	31.48	12.90	16.44	5.0e-05	0.009	91.5	0.589	0.06

Table S5. United Soil Surface Temperature Influence Index (UTII) calculated for weighted Unifrac distances between rhizosphere bacteria communities

Di	I^t_{i1}	I^t_{i2}	I^t_{i3}	I^t_i
D1(D1)	0.555	0.333	0.333	0.407
D2(D2)	0.111	0.111	0.333	0.185
D3(D4)	0.333	0.111	-0.333	0.037
D4(D5)	-0.555	0.111	0.555	0.037
D5(D6)	0.999	0.999	0.777	0.925
D6(D7)	-0.111	0.111	-0.111	-0.037
D7(D8)	-0.333	-0.111	-0.333	-0.259
D8(D9)	-0.333	0.111	0.111	-0.037
D9(D11)	0.111	0.333	0.111	0.185
D10(D12)	-0.555	-0.333	-0.111	-0.333

I^t_{i1} - Soil Surface Temperature Influence Index on the rhizosphere bacteria communities' weighted Unifrac distances in December 2017; I^t_{i2} is a similar index in January 2018; I^t_{i3} isa similar index in February 2018, I^t_i - United Soil Surface Temperature Influence Index on rhizosphere bacteria communities' weighted Unifrac distances in the 2017/18 season ($I^t_i = (I^t_{i1} + I^t_{i2} + I^t_{i3})/3$)

Table S6 – KEGG orthologs screened in predicted metabolic functions of rhizosphere microbiomes

Product	Enzymes involved in product synthesis	KEGG Orthologs
ACC deaminase	1-aminocyclopropane-1-carboxylate deaminase;<br>1-aminocyclopropane-1-carboxylate endolyase;<br>1-aminocyclopropane carboxylic acid deaminase	K01505
Indolacetic acid	ALDH; aldehyde dehydrogenase (NAD+)	K00128
	ALDH9A1; aldehyde dehydrogenase family 9 member A1	K00149
	ALDH7A1; aldehyde dehydrogenase family 7 member A1	K14085
	indole-3-acetate monooxygenase;<br>iacA (gene name)	K22027
	amidase;<br>acylamidase;<br>acylase (misleading);<br>amidohydrolase (ambiguous);<br>deaminase (ambiguous);<br>fatty acylamidase;<br>N-acetylaminohydrolase (ambiguous)	K01426
	indoleacetate---lysine synthetase;<br>indoleacetate:L-lysine ligase (ADP-forming)	K03397
	benzaldehyde dehydrogenase (NAD+) / indole-3-acetaldehyde oxidase	K22417
	indole-3-acetaldehyde oxidase	K11817
	indole-3-acetate O-methyltransferase;<br>IAA carboxylmethyltransferase;	K18848
	indole-3-acetate beta-glucosyltransferase;<br>uridine diphosphoglucose-indoleacetate glucosyltransferase;<br>UDPG-indol-3-ylacetyl glucosyl transferase;<br>UDP-glucose:indol-3-ylacetate glucosyltransferase;<br>indol-3-ylacetylglucose synthase;<br>UDP-glucose:indol-3-ylacetate glucosyl-transferase;<br>IAGlu synthase;<br>IAA-glucose synthase;<br>UDP-glucose:indole-3-acetate beta-D-glucosyltransferase	K13692
	nitrilase;<br>acetonitrilase;<br>benzonitrilase	K01501
	indole-3-pyruvate monooxygenase;<br>YUC2 (gene name);<br>spi1 (gene name)	K11816
	L-tryptophan---pyruvate aminotransferase;<br>TAA1 (gene name);<br>vt2 (gene name	K16903
	indoleacetate decarboxylase;<br>IAD	K23384
	tryptophan 2-monooxygenase;<br>tms1 (gene name);<br>iaaM (gene name)	K00466
	indolepyruvate decarboxylase	K04103

Product	Enzymes involved in product synthesis	KEGG Orthologs
Siderophore	L-lysine 6-monooxygenase	K03897
	N6-hydroxylysine N-acetyltransferase	K03896
	citrate:N6-acetyl-N6-hydroxy-L-lysine ligase	K03894
	aerobactin synthase	K03895
	salicylate biosynthesis isochorismate synthase	K01851
	menaquinone-specific isochorismate synthase	K02552
	isochorismate synthase	K02361
	phosphopantetheinyl transferase,	K06133
	bifunctional isochorismate lyase / aryl carrier protein	K01252
	isochorismatase	K01252
	2,3-dihydro-2,3-dihydroxybenzoate dehydrogenase	K00216
	(2,3-dihydroxybenzoyl)adenylate synthase, 2,3-dihydroxybenzoate-[DhbB aryl-carrier protein] ligase, enterobactin synthase	K02362
	enterobactin synthetase component D	K02362
	2,3-dihydroxybenzoate-AMP ligase	K02363
	enterobactin synthetase component F	K02364
	vibriobactin synthetase	K04778
	nonribosomal peptide synthetase VibF	K12237
	nonribosomal peptide synthetase DhbF	K04780
	nonribosomal peptide synthetase MxcG	K15653
	aminotransferase MxcL	K15681
	salicylate synthetase	K04781
	isochorismate pyruvate lyase	K04782
	pyochelin biosynthesis protein PchD	K12238
	dihydroaeruginoic acid synthetase	K12239
	pyochelin synthetase	K12240
	pyochelin biosynthetic protein PchG	K12241
	yersiniabactin salicyl-AMP ligase	K04783
	yersiniabactin nonribosomal peptide synthetase	K04784
	yersiniabactin synthetase, thiazolinyl reductase component	K04785
	yersiniabactin nonribosomal peptide/polyketide synthase	K04786
	mycobactin salicyl-AMP ligase	K04787
	mycobactin phenyloxazoline synthetase	K04788
	mycobactin peptide synthetase MbtE	K04789
	mycobactin polyketide synthetase MbtC	K04790
	mycobactin polyketide synthetase MbtD	K04791
	mycobactin peptide synthetase MbtF	K04792
	mycobactin lysine-N-oxygenase	K04793

Table A2 - List of prokaryotic taxa found in the rhizosphere microbial communities of D. antarctica, C. quitensis and D. caespitosa; ND -no data

Phylum	Class	Order	Family	Genus
[Thermi]	Deinococci	Deinococcales	Deinococcaceae	Deinococcus
			Trueperaceae	Truepera
		Thermales	Thermaceae	Meiothermus
Acidobacteria	[Chloracidobacteria]	Лис.24	ND	ND
		DS-100	ND	ND
		PK29	ND	ND
		RB41	Ellin6075	ND
	Acidobacteria-5	ND	ND	ND
	Acidobacteria-6	CCU21	ND	ND
		iii1-15	mb2424	ND
			RB40	ND
		ND	ND	ND
	Acidobacteriia	Acidobacteriales	Acidobacteriaceae	Acidicapsa
				Granulicella
			Koribacteraceae	Candidatus Koribacter
			ND	ND
	DA052	Ellin6513	ND	ND
	iii1-8	32-20	ND	ND
		DS-18	ND	ND
		SJA-36	ND	ND
	PAUC37f	ND	ND	ND
	RB25	ND	ND	ND
	Solibacteres	Solibacterales	[Bryobacteraceae]	Bryobacter
			PAUC26f	ND
			Solibacteraceae	Candidatus Solibacter
			ND	ND
	Sva0725	Sva0725	ND	ND
	TM1	ND	ND	ND
			AKIW874	ND

Actinobacteria	Acidimicrobiia	Acidimicrobiales	C111	ND
			EB1017	ND
			Iamiaceae	Iamia
			JdFBGBact	ND
			Microthrixaceae	Candidatus Microthrix
	Actinobacteria	Actinomycetales	ACK-M1	ND
			Actinosynnemataceae	ND
			Cellulomonadaceae	Cellulomonas
				Demequina
			Corynebacteriaceae	Corynebacterium
			Dermabacteraceae	Brachybacterium
			Dermacoccaceae	Dermacoccus
			Dermatophilaceae	ND
			Dietziaceae	Dietzia
			Frankiaceae	ND
			Geodermatophilaceae	Blastococcus
			Gordoniaceae	Gordonia
			Intrasporangiaceae	Terracoccus
				Phycicoccus
			Jonesiaceae	ND
			Kineosporiaceae	Kineococcus
				Kineosporia
			Microbacteriaceae	Candidatus Aquiluna
				Cryocola
				Frigoribacterium
				Leucobacter
				Salinibacterium
			Micrococcaceae	Arthrobacter
			Micromonosporaceae	Catellatospora
				Couchioplanes
				Dactylosporangium
				Pilimelia
			Mycobacteriaceae	Mycobacterium

			Nakamurellaceae	ND
			Nocardiaceae	Rhodococcus
				Nocardia
			Nocardioidaceae	Aeromicrobium
				Nocardioides
				Kribbella
			Propionibacteriaceae	ND
			Pseudonocardiaceae	Pseudonocardia
				Actinomycetospora
			Sanguibacteraceae	Sanguibacter
			Sporichthyaceae	ND
				Streptomyces
			Streptosporangiaceae	Streptosporangium
	MB-A2-108	0319-7L14	ND	ND
	Nitriliruptoria	Euzebyales	Euzebyaceae	Euzebya
	Rubrobacteria	Rubrobacterales	Rubrobacteraceae	Rubrobacter
	Thermoleophilia	Gaiellales	AK1AB1_02E	ND
			Gaiellaceae	ND
		Solirubrobacterales	Conexibacteraceae	Conexibacter
			Patulibacteraceae	Patulibacter
			Solirubrobacteraceae	Solirubrobacter
AD3	JG37-AG-4	ND	ND	ND
Armatimonadetes	[Fimbriimonadia]	[Fimbriimonadales]	[Fimbriimonadaceae]	Fimbriimonas
		ND	ND	ND
	0319-6E2	ND	ND	ND
	Armatimonadia	Armatimonadales	Armatimonadaceae	ND
		FW68	ND	ND
	Chthonomonadetes	Chthonomonadales	Chthonomonadaceae	ND
		SJA-22	ND	ND
	SHA-37	ND	ND	ND
	[Rhodothermi]	[Rhodothermales]	Rhodothermaceae	Rubricoccus
			Chitinophagaceae	Niabella
				Flavisolibacter

Bacteroidetes	[Saprospirae]	[Saprospirales]		Flavihumibacter
			Saprospiraceae	Aquirestis
				Lewinella
	At12OctB3	ND	ND	ND
	Bacteroidia	Bacteroidales	[Odoribacteraceae]	Odoribacter
			Bacteroidaceae	Bacteroides
			Marinilabiaceae	ND
			Porphyromonadaceae	Parabacteroides
				Macellibacteroides
			Rikenellaceae	Alistipes
			SB-1	ND
	BME43	ND	ND	ND
	Cytophagia	Cytophagales	[Amoebophilaceae]	ND
			Cyclobacteriaceae	Algoriphagus
			Cytophagaceae	Acidovorax
				Adhaeribacter
				Cytophaga
				Dyadobacter
				Emticicia
				Flectobacillus
				Flexibacter
				Hymenobacter
				Larkinella
				Leadbetterella
				Persicitalea
				Pontibacter
				Rhodocytophaga
				Rudanella
				Siphonobacter
				Spirosoma
				Sporocytophaga
			Flammeovirgaceae	Reichenbachiella
				Marinoscillum

				Flexibacter
	Flavobacteriia	Flavobacteriales	[Weeksellaceae]	Cloacibacterium
				Chryseobacterium
			Cryomorphaceae	Crocinitomix
				Cryomorpha
				Fluviicola
			Flavobacteriaceae	Aequorivita
				Arenibacter
				Flavobacterium
				Gelidibacter
				Winogradskyella
	SM1A07	ND	ND	ND
	Sphingobacteriia	Sphingobacteriales	Sphingobacteriaceae	Mucilaginibacter
				Pedobacter
				Sphingomonas
	VC2_1_Bac22	ND	ND	ND
BRC1	PRR-11	ND	ND	ND
Chlamydiae	Chlamydiia	Chlamydiales	Parachlamydiaceae	Candidatus Protochlamydia
				Parachlamydia
			Rhabdochlamydiaceae	Candidatus Rhabdochlamydia
Chlorobi	BSV26	PK329	ND	ND
		A89	ND	ND
	Ignavibacteria	Ignavibacteriales	Ignavibacteriaceae	ND
	OPB56	ND	ND	ND
	SJA-28	ND	ND	ND
	Anaerolineae	SBR1031	A4b	ND
			oc28	ND
			SJA-101	ND
		Anaerolineales	Anaerolinaceae	T78
				SHD-231
		Ardenscatenales	Ardenscatenaceae	Ardenscatena
		Caldilineales	Caldilineaceae	Caldilinea
				ND

Chloroflexi		A31	S47	ND
		H39	ND	ND
	C0119	ND	ND	ND
	Chloroflexi	[Roseiflexales]	[Kouleothrixaceae]	Rhodanobacter
				ND
			[Roseiflexaceae]	ND
		Chloroflexales	Chloroflexaceae	Chloronema
			FFCH7168	ND
			Oscillochloridaceae	Oscillochloris
	Ellin6529	ND	ND	ND
	Gitt-GS-136	ND	ND	ND
	Ktedonobacteria	Ktedonobacterales	Ktedonobacteraceae	ND
		Thermogemmatisporales	Thermogemmatisporaceae	ND
		B10-SB3A	ND	ND
	S085	ND	ND	ND
	SHA-26	ND	ND	ND
	Thermomicrobia	JG30-KF-CM45	ND	ND
	TK10	B07_WMSP1	fFFCH4570	ND
		AKYG885	Dol23	ND
			5B-12	ND
		mle1-48	ND	ND
Crenarchaeota	Thaumarchaeota	Cenarchaeales	SAGMA-X	ND
			Cenarchaeaceae	Nitrosopumilus
		Nitrososphaerales	Nitrososphaeraceae	Candidatus Nitrososphaera
				Pseudoxanthomonas
Cyanobacteria	4C0d-2	MLE1-12	ND	ND
	ML635J-21	ND	ND	ND
	Nostocophycideae	Nostocales	Nostocaceae	Nostoc
				Trichormus
				Cylindrospermopsis
	Oscillatoriophycideae	Chroococcales	Microcystaceae	Microcystis
			Xenococcaceae	ND
		Oscillatoriales	Phormidiaceae	Phormidium

	Synechococcophycideae	Pseudanabaenales	Pseudanabaenaceae	Leptolyngbya
				Pseudanabaena
		Synechococcales	Chamaesiphonaceae	ND
			Synechococcaceae	Synechococcus
Elusimicrobia	Elusimicrobia	FAC88	ND	ND
	Endomicrobia	ND	ND	ND
Euryarchaeota	Thermoplasmata	E2	[Methanomassiliicoccaceae]	ND
	Methanobacteria	Methanobacteriales	Methanobacteriaceae	Methanobrevibacter
FBP	ND	ND	ND	ND
FCPU426	ND	ND	ND	ND
Fibrobacteres	Fibrobacteria	258ds10	ND	ND
Firmicutes	Bacilli	Bacillales	[Exiguobacteraceae]	Exiguobacterium
			Thermoactinomycetaceae	ND
			Alicyclobacillaceae	Alicyclobacillus
			Bacillaceae	Virgibacillus
				Bacillus
			Paenibacillaceae	Paenibacillus
			Planococcaceae	Sporosarcina
				Planomicrobium
			Staphylococcaceae	Staphylococcus
		Lactobacillales	Carnobacteriaceae	Carnobacterium
			Enterococcaceae	Enterococcus
			Aerococcaceae	ND
			Streptococcaceae	Streptococcus
	Clostridia	Clostridiales	[Tissierellaceae]	ND
			Christensenellaceae	ND
			Clostridiaceae	Proteiniclasticum
				Clostridium
				Acetobacterium
			Lachnospiraceae	Roseburia
				Clostridium
			Peptococcaceae	Desulfosporosinus
			Peptostreptococcaceae	ND

			Ruminococcaceae	Faecalibacterium
				Ruminococcus
			Veillonellaceae	Megamonas
		SHA-98	D2	ND
				Erysipelothrix
				Catenibacterium
				Bulleidia
Fusobacteria	Fusobacteriia	Fusobacteriales	Leptotrichiaceae	Leptotrichia
			Fusobacteriaceae	Cetobacterium
Gemmatimonadetes	Gemm-1	ND	ND	ND
	Gemm-2	ND	ND	ND
	Gemm-3	ND	ND	ND
	Gemm-5	ND	ND	ND
	Gemmatimonadetes	Gemmatimonadales	A1-B1	ND
			Ellin5301	ND
			Gemmatimonadaceae	Gemmatimonas
		ND	ND	ND
GN02	3BR-5F	ND	ND	ND
	GKS2-174	ND	ND	ND
	BD1-5	ND	ND	ND
Nitrospirae	Nitrospira	Nitrospirales	Nitrospiraceae	Nitrospira
				JG37-AG-70
			0319-6A21	ND
NKB19	TSBW08	ND	ND	ND
OD1	Mb-NB09	ND	ND	ND
	SM2F11	ND	ND	ND
	ZB2	ND	ND	ND
OP11	OP11-3	ND	ND	ND
	OP11-4	ND	ND	ND
OP3	koll11	ND	ND	ND
	PBS-25	ND	ND	ND
	[Brocadiae]	Brocadiales	Brocadiaceae	Candidatus Brocadia
	028H05-P-BN-P5	ND	ND	ND

Planctomycetes	BD7-11	ND	ND	ND
	C6	d113	ND	ND
	OM190	agg27	ND	ND
	Phycisphaerae	Phycisphaerales	Phycisphaeraceae	ND
	Pla4	ND	ND	ND
	Planctomycetia	Gemmatales	Gemmataceae	Gemmata
			Isosphaeraceae	Isosphaera
				ND
		Pirellulales	Pirellulaceae	A17
				Blastopirellula
				Pirellula
				Rhodopirellula
		Planctomycetales	Planctomycetaceae	Planctomyces
		B97	ND	ND
	vadinHA49	DH61	ND	ND
			Acetobacteraceae	Acidiphilium
				Acidisoma
				Acidocella
				Roseococcus
				Roseomonas
			Rhodospirillaceae	Dongia
				Inquilinus
				Phaeospirillum
				Reyranella
				Skermanella
				Stella
			Aurantimonadaceae	ND
			Beijerinckiaceae	Beijerinckia
				Methylocella
			Bradyrhizobiaceae	Balneimonas
				Bosea
				Bradyrhizobium
				Nitrobacter

	Alphaproteobacteria	Rhizobiales	Hyphomicrobiaceae	Devosia
				Hyphomicrobium
				Parvibaculum
				Pedomicrobium
				Rhodoplanes
				ND
			Methylobacteriaceae	Methylobacterium
			Methylocystaceae	Methylopila
				Pleomorphomonas
			Phyllobacteriaceae	Mesorhizobium
				Aminobacter
			Rhizobiaceae	ND
				Rhizobium
				Kaistia
				Agrobacterium
				Afifella
			Xanthobacteraceae	Labrys
			ND	ND
		Caulobacterales	Caulobacteraceae	Arthrospira
				Asticcacaulis
				Brevundimonas
				Caulobacter
				Mycoplana
				Phenylobacterium
		Sphingomonadales	Erythrobacteraceae	Lutibacterium
				ND
			Sphingomonadaceae	Arthrobacter
				Kaistobacter
				Novosphingobium
				Sphingobium
				Sphingomonas
				Sphingopyxis
				Zymomonas

		Rhodobacterales	Hyphomonadaceae	Hyphomonas
				Woodsholea
			Rhodobacteraceae	Amaricoccus
				Loktanella
				Octadecabacter
				Paracoccus
				Rhodobacter
				Rubellimicrobium
		Rickettsiales	Rickettsiaceae	Rickettsia
				ND
	Betaproteobacteria	Burkholderiales	Alcaligenaceae	Achromobacter
				Sutterella
			Burkholderiaceae	Burkholderia
			Comamonadaceae	Acidovorax
				Aquabacterium
				Hydrogenophaga
				Hylemonella
				Inhella
				Leptothrix
				Methylibium
				Polaromonas
				Rhodoferax
				Simplicispira
			Oxalobacteraceae	Herminiimonas
				Janthinobacterium
				Massilia
				Polynucleobacter
			ND	ND
		A21b	UD5	ND
			EB1003	ND
		Gallionellales	Gallionellaceae	ND
		Hydrogenophilales	Hydrogenophilaceae	Thiobacillus
		Methylophilales	Methylophilaceae	Methylotenera

		Neisseriales	Neisseriaceae	Deefgea
		Nitrosomonadales	Nitrosomonadaceae	Nitrosovibrio
		Procabacteriales	Procabacteriaceae	Procabacter
		Rhodocyclales	Rhodocyclaceae	Uliginosibacterium
				Dechloromonas
				Candidatus Accumulibacter
	Deltaproteobacteria	[Entotheonellales]	[Entotheonellaceae]	ND
		Bdellovibrionales	Bacteriovoracaceae	Peredibacter
			Bdellovibrionaceae	Bdellovibrio
		Desulfovibrionales	Desulfovibrionaceae	Desulfovibrio
		Desulfuromonadales	Geobacteraceae	Geobacter
		NB1-j	JTB38	ND
			MND4	ND
			NB1-i	ND
		Myxococcales	Haliangiaceae	ND
			Cystobacteraceae	Cystobacter
				ND
			0319-6G20	ND
			Myxococcaceae	Corallococcus
				Anaeromyxobacter
			Nannocystaceae	Nannocystis
				Plesiocystis
			OM27	Arthrobacter
			Polyangiaceae	Aetherobacter
				Chondromyces
				Sorangium
		Syntrophobacterales	Syntrophaceae	Syntrophus
	Epsilonproteobacteria	Campylobacterales	Campylobacteraceae	Arcobacter
			[Chromatiaceae]	Rheinheimera
			125ds10	ND
			211ds20	ND
			Aeromonadaceae	Oceanisphaera
				Porticoccus

	Gammaproteobacteria	Alteromonadales	Alteromonadaceae	Cellvibrio
				Glaciecola
				Marinobacter
				HB2-32-21
			OM60	ND
			Psychromonadaceae	Psychromonas
			HTCC2188	HTCC
		Legionellales	Coxiellaceae	Aquicella
				Rickettsiella
			Legionellaceae	Legionella
		Pseudomonadales	Moraxellaceae	Psychrobacter
				Acinetobacter
				Perlucidibaca
				Alkanindiges
			Pseudomonadaceae	Pseudomonas
		Oceanospirillales	Halomonadaceae	Halomonas
			Oceanospirillaceae	Neptunomonas
		Xanthomonadales	Sinobacteraceae	Steroidobacter
				Alkanibacter
				Nevskia
				Panacagrimonas
			Xanthomonadaceae	Arenimonas
				Dokdonella
				Gynumella
				Luteibacter
				Luteimonas
				Lysobacter
				Pseudoxanthomonas
				Rhodanobacter
				Stenotrophomonas
				Thermomonas
		Salinisphaerales	Salinisphaeraceae	Salinisphaera
		Chromatiales	Ectothiorhodospiraceae	ND

		Enterobacteriales	Enterobacteriaceae	Buchnera
		HTCC2188	HTCC2089	ND
		Vibrionales	Vibrionaceae	Photobacterium
		Pasteurellales	Pasteurellaceae	ND
		Thiotrichales	Piscirickettsiaceae	ND
		[Marinicellales]	[Marinicellaceae]	ND
	TA18	CV90	ND	ND
Spirochaetes	[Leptospirae]	[Leptospirales]	Leptospiraceae	Turneriella
				Leptonema
	Spirochaetes	Spirochaetales	Spirochaetaceae	Spirochaeta
SR1	ND	ND	ND	ND
Synergistetes	Synergistia	Synergistales	Synergistaceae	vadinCA02
			Dethiosulfovibrionaceae	HA73
Tenericutes	Mollicutes	Anaeroplasmatales	Anaeroplasmataceae	Asteroleplasma
		Entomoplasmatales	ND	ND
TM6	SJA-4	ND	ND	ND
	SBRH58	ND	ND	ND
TM7	SC3	ND	ND	ND
	TM7-3	EW055	ND	ND
	TM7-1	ND	ND	ND
	MJK10	ND	ND	ND
Verrucomicrobia	[Spartobacteria]	[Chthoniobacterales]	[Chthoniobacteraceae]	_heteroC45heteroC45_4W
				Candidatus Xiphinematobacter
				Chthoniobacter
				DA101
				Ellin506
				OR-59
			01D2Z36	ND
	Opitutae	Opitutales	Opitutaceae	Opitutus
		[Cerasicoccales]	[Cerasicoccaceae]	ND
	[Methylacidiphilae]	Methylacidiphilales	LD19	ND
			[Pedosphaeraceae]	Pedosphaera
			auto67_4W	ND

	[Pedosphaerae]	[Pedosphaerales]	Ellin515	ND
			Ellin517	ND
			R4-41B	ND
	Verruco-5	LD1-PB3	ND	ND
	Verrucomicrobiae	Verrucomicrobiales	Verrucomicrobiaceae	Luteolibacter
				Prosthecobacter
				Verrucomicrobium
WPS-2	ND	ND	ND	ND
WS3	PRR-12	Sediment-1	PRR-10	ND
WWE1	[Cloacamonae]	[Cloacamonales]	[Cloacamonaceae]	W22
